# Supplementary figures and images for: Endemicity of Paragonimus and paragonimiasis in Sub-Saharan Africa: A systematic review and mapping reveals stability of transmission in endemic foci for a multi-host parasite system
Source: PLoS Negl Trop Dis. 2021 Feb 5;15(2):e0009120. doi: 10.1371/journal.pntd.0009120 (PMC7891758; doi:10.1371/journal.pntd.0009120)

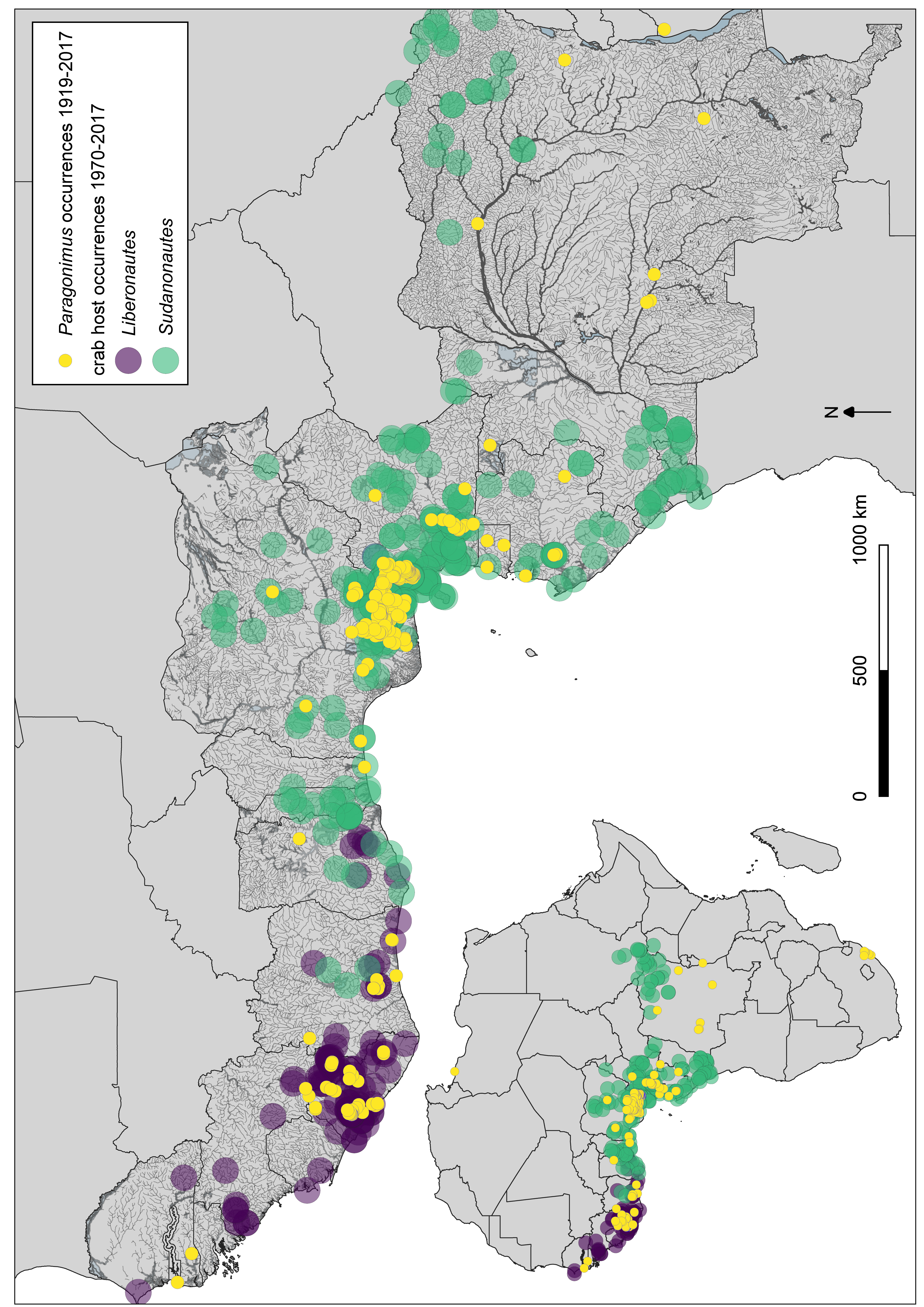

Supplement: S1 Fig — Data compiled from the literature (S5 Table) and an the occurrence dataset from NC (S6 Table). (TIF) [file pntd.0009120.s001.tif]
