# Supplementary material for: Endemicity of Paragonimus and paragonimiasis in Sub-Saharan Africa: A systematic review and mapping reveals stability of transmission in endemic foci for a multi-host parasite system
Source: PLoS Negl Trop Dis. 2021 Feb 5;15(2):e0009120. doi: 10.1371/journal.pntd.0009120 (PMC7891758; doi:10.1371/journal.pntd.0009120)
Supplement: Attachment — Submitted filename: Rabone_etal_SR+mapping_para_in_Africa_020-09-09_AL_Revs_3Oct2020.pdf [file pntd.0009120.s009.pdf]

REVIEW

Endemicity of *Paragonimus* and paragonimiasis in West and Central Africa, a systematic review and mapping reveals stability of transmission in endemic foci for a multi-host parasite system

Muriel Rabone[1, 2*], Joris Wiethase[3], Paul F. Clark[1], David Rollinson[1,2], Neil Cumberlidge[4], Aidan M. Emery[1,2]

1 Department of Life Sciences, Natural History Museum, Cromwell Road, London SW7 5BD, United Kingdom, **2** London Centre for Neglected Tropical Disease Research, School of Public Health, Faculty of Medicine, Imperial College London, Norfolk Place, London W2 1PG, United Kingdom, **3** Department of Biology, University of York, Wentworth Way, York, YO10 5DD, United Kingdom, **4** Department of Biology, Northern Michigan University, 1401 Presque Isle Avenue, Marquette, Michigan 49855, United States of America

[*] m.rabone@nhm.ac.uk

ORCID IDs:
MER: 0000-0002-8351-2313
JW: 0000-0003-2008-1613
PC: 0000-0001-6862-3982
DR: 0000-0003-1999-1716
NC: 0000-0002-0568-6414
AME: 0000-0001-9028-8586

Running header: Rabone et al.; Endemicity of paragonimiasis in West and Central Africa

Key Words: paragonimiasis; *Paragonimus uterobilateralis*; *Paragonimus africanus*; human lung fluke disease; foodborne disease/foodborne trematodes; *Sudanonautes*; *Liberonautes*; Nigeria, Cameroon, West and Central Africa; Georeferencing; mapping; endemic foci

Abstract

Paragonimiasis is caused by zoonotic trematodes of *Paragonimus* spp., found in Asia, the Americas and Africa, particularly in tropical regions. These parasites have a complex, multi-host life cycle, with mammalian definitive hosts and larval stages cycling through two intermediate hosts (freshwater snails and crabs). In Africa, paragonimiasis is particularly neglected, and remains the only human parasitic disease without a fully characterised life cycle. However Paragonimiasis has potentially significant impacts on public health in Africa, and prevalence has likely been underestimated through systemic under-reporting and misdiagnosis as tuberculosis due to a similar clinical presentation. We identified the need to synthesise current knowledge and map endemic foci for African *Paragonimus* spp. together with *Poikilorchis congolensis*, a rare, taxonomically distant trematode with a similar distribution and morphology. We present the first systematic review of African paragonimiasis, combined with mapping of all reported occurrences of *Paragonimus* spp. throughout Africa, from the 1910s to the present. In human surveys, numerous reports of significant recent transmission in Southeast Nigeria were uncovered, with high prevalence and intensity of infection. Overall prevalence was significantly higher for *P. uterobilateralis* compared to *P. africanus* across studies. The potential endemicity of *P. africanus* in Cote d'Ivoire is also reported. In freshwater crab intermediate hosts, differences in incidence, prevalence and intensity of either *P. uterobilateralis* or *P. africanus* were evident across genus and species, suggesting differences in susceptibility. Mapping showed temporal stability of endemic foci, with the majority of known occurrences of *Paragonimus* found in the rainforest zone of West and Central Africa, but with several outliers elsewhere on the continent. This suggests substantial under sampling and localised infection where potential host distributions overlap. Our review highlights the urgent need for increased sampling in active disease foci in Africa, particularly using molecular analysis to fully characterise *Paragonimus* species and their hosts.

AUTHOR SUMMARY

Paragonimiasis is caused by food-borne zoonotic trematodes of *Paragonimus* spp., multi-host parasites whose complex life cycle includes primary (snail), secondary (freshwater crab), and mammalian definitive hosts. In Africa, paragonimiasis is a neglected tropical disease, where prevalence has likely been underestimated through systemic under reporting and misdiagnosis as tuberculosis; where it remains the only human parasite without a fully characterised life cycle. We present the first systematic review of the literature relating to paragonimiasis in Africa, including 142 publications. In human studies, we uncovered significant transmission in Southeast Nigeria, and recent transmission in Cote d'Ivoire and Cameroon in endemic foci; significantly higher prevalence of *P. uterobilateralis* than *P. africanus*; and evidence supporting the existence of *P. africanus* in Cote d'Ivoire. In freshwater crab intermediate hosts, incidence, prevalence and intensity of either *P. uterobilateralis* or *P. africanus* varied across genera and species, suggesting differences in susceptibility. Mapping revealed evidence of temporal stability at endemic foci in rainforest regions; and widespread outliers. Numerous reports of significant recent transmission, particularly in Southeast Nigeria should be heeded by the international NTD research community. Increased studies, in particular utilising molecular tools to ascertain the real distribution, and diversity of *Paragonimus* species in Africa are urgently needed.

Introduction

Paragonimiasis is caused by food-borne zoonotic trematodes of *Paragonimus* spp., multi-host parasites whose complex life cycle includes a primary (or first intermediate) snail host; a secondary (or second intermediate) decapod crustacean host, and a mammalian definitive host. Human paragonimiasis has a global distribution in the Americas, Africa, and Asia, particularly in tropical regions (1, 2). An estimated 23 million people are infected worldwide, with the greatest disease burden in Asia (1-3). The taxonomy of *Paragonimus* is problematic and in need of revision, but currently there are over 50 recognised species of *Paragonimus*, 7 of which cause disease in humans (1, 2). Paragonimiasis in humans is generally caused by the consumption of incompletely cooked freshwater crabs that harbour the infective metacercarial larval stages of the parasite, but it can also be acquired by eating uncooked paratenic hosts infected with juvenile worms in development stasis (1, 4). *Paragonimus* is a euryxenous parasite with numerous mammalian reservoir hosts, including mongooses, civets, primates and domestic dogs and cats in Africa for example (5-8). The parasite also develops

in a broad range of molluscan first intermediate hosts, such as Rissoidea and Cerithoidea in Asia (9), and a broad range of crustacean second intermediate hosts, mostly species of brachyuran crab found in freshwater habitats, some of which are semi-terrestrial (10). In terms of the clinical picture, paragonimiasis is primarily a disease of the lungs, where adult worms lodge in lung tissue, although ectopic infections are relatively common in some Asian species (2). The main clinical symptoms include persistent cough, hemoptysis, and abdominal or chest pain (3). With often overlapping symptoms, there is the potential for diagnostic confusion with tuberculosis (TB; 3, 11).

In Africa, paragonimiasis-like lung infections were first reported in the 1920s (12, 13), with sporadic reports in the following 3decades (14-18). The first species of *Paragonimus* were described in 1965: for *Paragonimus africanus* (7) and *Paragonimus uterobilateralis* (8). Paragonimiasis in Africa came to widespread attention with an outbreak in Southeast Nigeria in the late 1960s, stemming from food shortages and concomitant consumption of freshwater crabs as a result of the Nigerian Civil war of 1967-1970 (19-21). In more recent times, paragonimiasis in Africa has received remarkably little attention from the international research community, despite several recent studies reporting significant transmission from Nigeria in particular (22-27); and also Cameroon (28, 29). Paragonimiasis in Africa remains the only human parasite without a fully characterised life cycle, with the first intermediate snail hosts of the African species of *Paragonimus* unconfirmed, and the miracidia, rediae and cercarial stages undescribed. Suspected host snail species (co-occurring in known crab host habitats) include Achatinidae in Cameroon (30, 31); *Potadoma freethii* in Cameroon (7) and Nigeria (26, 32); *Melania* in Nigeria (33); and *P. sanctipauli*, *Afropomus balanoidea* (34); and *Homorus* (*Striosubulina*) *striateila* (Achatinidae) in Liberia (35). The latter studies in Liberia attempted experimental infections, but the candidate *Paragonimus*-like cercariae failed to infect known freshwater crab hosts (*Liberonautes*) collected at the disease foci (34, 36). The second intermediate hosts of *Paragonimus* in Africa are better known, with 8 species of freshwater crabs in 2 genera, *Sudanonautes*, and *Liberonautes* implicated as secondary hosts (10). However, current taxonomic revisions involving host species may alter the generic assignments of some taxa.

Four species of *Paragonimus* have been recorded from Africa to date: *P. uterobilateralis*, *P. africanus*, *P. gondwanensis*, and *P. kerberti* (7, 8, 37, 38). All species descriptions are based on morphology only, none have yet been characterised with molecular data, and only three sequences of one species have been published on GenBank (5.8S, 28S rRNA and ITS2 from *P. africanus*; 29, 39). Species of *Paragonimus* in Africa are therefore

currently distinguished by the morphological characters of egg and metacercariae. Aka et al. (40) suggested there may be undescribed species of *Paragonimus* in West and Central Africa, but the lack of molecular data is hampering the ability to answer this question, as well as the study of the life cycle and distribution of these parasites more generally.

Few attempts at spatial characterisation of paragonimiasis in Africa have been made since the mapping of freshwater crab hosts in endemic foci in Nigeria and Cameroon by Voelker & Sachs (41), despite the need to map endemic foci noted as early as 1972 by Nwokolo (20). Mapping is essential for highly focal diseases, for example recent studies illustrate the temporal stability of endemic foci in schistosomiasis (42). Foci of paragonimiasis may be similarly stable over time, given the likelihood of the relatively long persistence of mature metacercariae in freshwater crab hosts, and of adult flukes in the lungs of mammalian definitive hosts (1, 4). Although recent reviews have discussed the need to address the extensive knowledge gaps of paragonimiasis in Africa and update the true public health significance of the disease (43-46), no systematic review or meta-analysis has previously been undertaken. Consequently, the aims of the current study are: to undertake a systematic review of paragonimiasis in Africa (including reports of the distantly-related trematode, *Poikilorchis congolensis* with diagnostic overlap with *Paragonimus*), to synthesise current knowledge and to map endemic foci. The specific aims of the present study are: 1. to ascertain the level of recent disease transmission; 2. to map reported confirmed occurrences of both *Paragonimus* and the freshwater crab intermediate hosts; and 3. ultimately establish both the historical and recent distribution of this most neglected tropical disease throughout Africa.

Materials and methods

Ethics statement: The data included in this study were anonymised aggregated data from previously published studies only.

Study protocol: A systematic review was undertaken according to PRISMA guidelines (47). The *a priori* review question was as follows:

What is the evidence, extent and distribution of recent/ongoing transmission of paragonimiasis in Africa?

Eligibility criteria: Peer reviewed publications on African *Paragonimus* spp./paragonimiasis were included in all languages (included Dutch, English, French, German, Italian, and Japanese).

Information sources: Online databases were searched through the Natural History Museum (NHM) London library, including: Google Scholar, African Journals Online, PubMed, ScienceDirect, and Web of Science. Publications not openly accessible were requested through interlibrary loans, and ones unavailable by those means were sourced directly in some instances by contacting authors/researchers in the field (David Aka, David Blair, and Chris Appleton). All citations were comprehensively checked, and any additional publications included. References of all papers were also systematically checked and added if they had not previously been identified through database searches.

Period: The review was carried out from November 2018-June 2020 by authors MR and JW. The date of last search was the 20th June, 2020.

Search strategies: Search terms included: paragonimiasis, *Paragonimus*, *Paragonimus africanus/uterobilateralis/gondwanensis/kerberti*, Africa, for all databases. Additional searches including these terms and the following were made in combination searches: all country names for West and Central Africa (Nigeria, Cameroon, Cote d'Ivoire, Gabon, Equatorial Guinea, Ghana, Liberia, Guinea, Senegal, Central African Republic, Democratic Republic of the Congo/DRC, Republic of the Congo, Central African Republic, Benin, Togo, Sierra Leone, Senegal, Gambia and Burkina Faso); names of endemic regions, (Cross River, Imo River); prior country names to ensure historical records were found (Haute-Volta, Zaire); relevant French terms (paragonimoses and distomatoses), and intermediate host crab genera names: *Sudanonautes*, *Liberonautes*, *Potamonautes* and *Potamonemus*.

Following initial investigations, an additional review was undertaken for *Poikilorchis congolensis* (also known as *Achillurbainia congolensis*). This trematode is the monotypic species of *Poikilorchis* and assigned to Achillubainiidae Dolfus, 1939, which has only one

other species, *Achillurbainia nouveli* Dolfus, 1939 (48). While *P. congolensis* is taxonomically distant, early investigations showed confusion in identification, with similar egg morphology and overlapping endemism. Therefore the decision was made to include the species as a subset analysis. Symptomology of a characteristic extra-pulmonary localisation in the form of a retroauricular or neck cyst (48) never reported in paragonimiasis, and egg negative sputum samples, were assessed to be consistent with infection of *P. congolensis* only, as evidenced in the literature (49, 50). Search terms were as above, minus the crab genera names and replacing *Paragonimus* spp. with *Poikilorchis/congolensis*.

Study selection: Criteria for inclusion were publications on African paragonimiasis only, those addressing Asian or American paragonimiasis/*Paragonimus* species were excluded. All original research, including non-survey based works, for example case reports, experimental studies or questionnaire-only based studies were included. Any publications that did not contain new data, i.e., analysing the findings of an earlier study, were excluded. All papers meeting selection criteria were read in full by MR and JW, those in German however, solely by JW. Other papers not in English were translated in Google Translate. The sole paper in Japanese, which also had relevant sections in English, was checked by a native speaker. Reviews were excluded, but those on paragonimiasis in Africa were read in full and references checked against the main publication list and any new references added (43-46, 51, 52).

Data collection process: Authors MR and JW extracted data from the publications and cross checked a proportion of the other's data records. A database was created in Microsoft Excel 365 (S1 & 5 Tables). Alongside key publication details, data recorded included the following: country; year of study; category (e.g., case report, survey, and for latter, scope of survey = humans/freshwater crabs/freshwater snails or combinations of); *Paragonimus* species reported; a brief description of the study and key findings; what the paragonimiasis infection was described from/how it was diagnosed, e.g., eggs in sputum (S1 Table). Where present, clinical findings were also recorded, e.g., symptoms observed, and details for any other diseases screened for (e.g., TB, and soil transmitted helminths). Other data recorded included: prevalence measurements for any hosts surveyed; egg size measurements where reported; and parasite counts for analysing intensity of infection (egg or metacercariae numbers per host or adult worm counts for wild-caught mammals), as overall averages and individual counts where available. Additional tables for prevalence, intensity and egg-size measurement data were created to allow for the analysis and recording of individual measurements (S2-4 Tables). In many cases study period was not reported in the paper, here it was estimated as

two years prior to publication. In analyses of dates, decade was used to mitigate uncertainty around sampling period. For human cases, the period or year when symptoms first appeared was noted, if recorded. If locality maps or photographs of collected larval stages were included, this was recorded also. Additional data were sourced directly from authors if required for analysis (see Acknowledgements). All publications, both included and excluded from analysis, are listed in S1 Table.

Confirmation of infection: Identification of *Paragonimus* in the literature was generally undertaken by microscopy, i.e., the microscopic identification of eggs or metacercariae. Only 4 studies used molecular analysis and/or serology (29, 39, 53, 54). Here, identification was accepted on microscopy only, this criterion was applied even if the result was contrary to the diagnosis in the paper, e.g., a positive case based on serology results or symptoms only was recorded here as unconfirmed. This assessment was made for consistency and to ensure that only current infections were identified. As a result, 11 cases based solely on serology, and 3 on symptoms only were recorded as unconfirmed and excluded from the analysis. Positive molecular identification was not a suitable criterion for exclusion having been utilised in only 2 studies (29, 39). A study that reported parasite eggs morphologically consistent with *Paragonimus*, but with PCR amplification unsuccessful was therefore considered a confirmed case for this review (55). This author also retrospectively evaluated an earlier survey with an assessment of "*Paragonimus*-like" infections as positive for *Paragonimus* based on the available evidence (56), we concurred with the assessment. For *Poikilorchis congolensis*, 1 case was diagnosed as caused by either *Poikilorchis* or *Paragonimus* (57) but with symptoms of egg-negative sputum and the characteristic retroauricular cyst consistent only with *Poikilorchis*; it was assessed to be the latter (the study authors made a similar observation). Some early publications mistakenly noted *Poikilorchis* cases as *Paragonimus* but these were corrected in later publications for the same reason (a retroauricular or neck cyst containing *Poikilorchis* eggs being present and absence of eggs in sputum), and the same assessment was made for this review. In cases where *Paragonimus* eggs were isolated from stool only, any available photographs were assessed, and evidence of positive identification of other parasite species checked to rule out the possibility of misidentification of another trematode (none were found).

Data analysis and summary statistics: All data were analysed in R version 3.5.3 (2019-03-11) "Great Truth". The majority of the literature in the systematic review was highly heterogeneous and a qualitative review was considered the best approach for analysis. A subset of publications was selected for a comparison of prevalence and intensity across

studies, separately for each host category (detailed below). Averages and standard deviation values were computed in R, using the tidyverse package (58). Weighted means were computed, for example, egg measurement averages in a given study weighted by total eggs measured; average intensity, weighted by total number of infected hosts (per host-category) per study, and in prevalence, by total surveyed for the study (Table 1). The statistical significance of differences across studies between groups, for example in prevalence and intensity of infected hosts were tested with a Wilcoxon rank sum test, which does not assume a normal distribution, given the high heterogeneity and variance among studies. All statistical tests were conducted with the significance level $\alpha = 0.05$ for rejecting null hypotheses.

Prevalence: In human surveys, publications were excluded if the study population was selected from people pre-determined as likely infection candidates, for example hospital surveys of pulmonary clinic patients. A publication was also excluded if the total study population was not given. In some papers, study populations were identified and subsequently only those individuals presenting symptoms were recruited for the survey. These publications were included as the original study population total was also given. Both cross-sectional (community-wide) and school-based surveys were included. Prevalence was recorded for all hosts: human/other mammals, and intermediate freshwater crab hosts. Prevalence for crabs was calculated by species, excluding *Callinectes marginatus*, regarded as an unconfirmed host (59; NC, pers. comm.). Prevalence differences by demographics (sex and age) were also examined. For sex, both overall total and proportion of female/male patients positive were recorded, and whether any difference was significant, if reported. For age data, the defined age categories, and proportion of prevalence per category were recorded. Age categories were generally in 5- or 10-year divisions (e.g., 5-10, 11-15, 16-20 year olds), but varied by study population and publication (sometimes totals were not given or reported as region sub-totals). Because of this high heterogeneity, overall findings were aggregated to 10 year periods, and also summarised as general observations (S2 Table).

Intensity: Range and average for intensity of infection (egg/metacercariae count per host) were recorded, and the average over the study calculated if not reported. Generally, eggs were recorded per 5 ml of sputum (e/5g), or eggs per gram of stool (epg). In one case where egg counts were reported for 1ml sputum samples, this was recorded and counts adjusted to per 5ml to allow for comparability. In another publication, sputum samples of 1-5ml were collected, and this study was excluded from analysis. The sample preparation method was recorded, for example if the sputum sample was concentrated (centrifuged, generally with NaOH), or stool processing details, which generally involved a concentration and

sedimentation technique (slide preparations used in two cases). Details on crab dissection and screening for infection were also recorded. Generally tissue was homogenised and washed, and resulting sediment and supernatant screened for metacercariae, and metacercariae counts per host recorded. In 1 study, only the haemocoel was screened (60). Studies that also screened for TB used Ziehl Nielsen stain for Acid Fast Bacilli (AFB) for diagnosis. If multiple samples were collected per host and the average reported, this was recorded. In 3 publications, intensity was reported only by level (in 5ml sputum), i.e., low, medium or high, very high (generally 0-50, 51-100, 101>), these totals were not included as direct comparison was not possible with average egg count totals, but the levels reported were assessed in general observations. Egg count measurements from stool (in addition to sputum) reported in 3 studies were excluded for consistency. No experimental infections were included in the subset analysis for intensity or prevalence, but measurements of eggs isolated from experimental hosts were included in egg measurement analysis and the host details recorded (S3 Table).

Mapping

Data collection: Occurrence data (each unique sampling event; site and timepoint) were collected and assessed for mapping (S5 Table). All occurrences were recorded individually from each publication meeting criteria for inclusion in the study, e.g., a case report confirmed with evidence of eggs in sputum. No experimental studies were included, unless field collected data were also present. Locality data were also extracted from Cumberlidge et al. (Table 2; 43) and validated against the literature. Occurrence data collected included: year/s study was undertaken; country; region (verbatim) site name; verbatim site coordinates (if present); site type (village, school, hospital, crab collection site: river/stream or crab market); *Paragonimus* species, life stage, host species/taxonomy, totals of host surveyed and number infected per site, if recorded. Contextual notes on the occurrence, the locality and the infection in question were also made. Absence data for *Paragonimus* spp. were recorded if present, e.g., if a crab survey did not find infected crabs at a given site. Additional spatial data sources included river and waterway shapefiles, downloaded from DIVA-GIS (http://www.diva-gis.org/; 61); and crab intermediate host occurrences (unpublished data from author NC).

Inclusion criteria: Records with a confirmed life stage of *Paragonimus* spp. (e.g., eggs in sputum or metacercariae in crabs) were mapped, in the absence of other diagnostic markers for the disease. Six publications not in the systematic review were included in mapping,

either where locality data were more comprehensive than a duplicate publication that was included in the review (62); or where localities were referenced in another publication, but the primary text was unavailable (63-67). If the same occurrence was referenced in separate papers, the 'subsequent source' of the occurrence was also recorded. Any occurrences additional to the main data in a study (separate surveys, case reports or personal communications) not reported elsewhere, were recorded, with the publication in which they were cited as the relevant reference, e.g., Richard Bradbury pers. comm. in Morter et al. (68).

Georeferencing: Coordinates included in a publication were recorded and transformed to decimal degrees. Non georeferenced localities were located with Google Maps or the place-names database geonames.org, and the data source noted. If the locality name was not found, gazetteers were checked at the NHM library, and paper maps were scanned and checked for site names and geographical details. In some cases localities could not be georeferenced. Where localities or endemic zones were plotted on site maps in a publication, they were mapped using the GDAL georeferencer for QGIS, Version 3.10, Coruña (QGIS.org, 2020), and collated. In Voelker & Sachs (41), a large number of crab occurrences were included in 4 published maps, all were georeferenced using GDAL, and site names and locations on maps cross-referenced with Google Maps. Post georeferencing, current region and district names were recorded as well as verbatim ones given in publication, by a spatial join in R to records from GADM version 3.6, the database of GADM database of Global Administrative Areas accessed through the GADM portal at https://gadm.org/.

Uncertainty assessment: Many records, historical and recent, had a high degree of coordinate uncertainty. Contextual notes were made, and transformed to a category to try to assess uncertainty in a consistent and semi-structured way. For example, a case report where the patient lived in an endemic disease focus and had never lived elsewhere would be assessed as most likely a case of local transmission and a low uncertainty; vice versa for a patient who had also travelled to different regions and therefore could have obtained the infection elsewhere. Uncertainty was also assessed by site category. Hospitals were recorded as high uncertainty as patients in endemic regions may have travelled considerable distances to the nearest clinic or hospital, far from the site of transmission (19). If the village/town a patient was from was not recorded, the hospital locality was used. Schools were recorded as low uncertainty, with the balance of probability that the transmission site was local and that school children were unlikely to have lived previously in other endemic regions. For crab collection sites, crab markets were assessed as low uncertainty for markets in rainforest zones where crabs are generally sold live and fished locally, within a 20 km radius for example

(NC, pers. comm.). In urban markets however, crabs may be transported, sometimes even across borders (69), and these markets were recorded as high uncertainty. Direct crab collection sites, i.e., crab fishing sites in rivers/streams, were recorded as low uncertainty, as this represents direct sampling of the crab habitat; crabs do not move far along a watercourse, reproduce by direct development and have a small home range, although limited seasonal movements may occur with high water conditions (NC, pers. comm.). Both hospitals and crab markets were classified as 'indirect' sites, potentially representing a large catchment area, particularly hospitals. These low uncertainty localities were marked out separately on maps, e.g., *Paragonimus* life stages isolated from crab hosts or non-human mammal hosts (excluding domestic animals).

Results

Study selection: A total of 874 studies were initially recovered from databases, 169, Google scholar; 170, Pubmed; 64, Africa Journals Online; 362, Science Direct; 33, Web of Science; and an additional 76 from other sources, reference lists, and scanning journal holdings in NHM library (Fig 1). After removal of duplicates, 512 titles were screened, of which 205 full texts were identified, where 17 were unavailable and 39 were excluded with reasons given (S1 Table). This resulted in a final total of 142 studies selected for the systematic review, with all databases above represented apart from Science Direct (and all 76 publications from other sources), and 7 for the subset review of *Poikilorchis congolensis*.

General findings/study characteristics: Of the 142 scientific publications selected, 132 were peer-reviewed publications from 73 journals, and the remainder consisted of 3 book chapters, 2 thesis copies, 3 conference papers, 4 publications from grey literature such as reports/government publications, and 1 in preparation. One journal was particularly represented with 25 publications (Tropical Medicine and Parasitology, formerly Tropenmedizin und Parasitologie). For *Poikilorchis congolensis*, 7 publications were selected, all published in peer-reviewed journals.

Species: The number of studies by species was as follows: *Paragonimus uterobilateralis* (44), *P. africanus* (15), *P. africanus* and *P. uterobilateralis* together (8), and 1 each for *P. kerberti* and *P. gondwaniensis*. For 68 studies, the species was recorded here as *Paragonimus* sp. because the species was either not stated or incorrectly recorded as *P. westermani*. Two surveys found no evidence of *Paragonimus*, but was included as absence data (68, 70), and the remaining 3 publications were recorded here as Trematoda as were solely observations of microcercous cercariae in snails, regarded as unconfirmed (30, 31, 36; S1 Table). There were no reports of mixed infection in humans. Although one study from Southwest Cameroon reported eggs of *P. africanus* and *P. uterobilateralis* from patients, it was not recorded if any patient was expelling both species (71). Mixed infections were reported from crabs in one study, also from Cameroon (41). In 44 publications high magnification photos of parasite eggs/metacercariae were included. In total, the publications reported ca. 6400 cases of infection with *Paragonimus* spp. (freshwater crab hosts and human and reservoir mammalian hosts combined across studies), and ca. 4800 cases in humans only. For *Poikilorchis congolensis*, 7 publications recorded 11 cases in total.

Locations: The majority of studies were from 4 countries: Nigeria, 52; Cameroon, 27; Liberia, 14; and Cote d'Ivoire, 11 (Fig 2). *Paragonimus africanus* was recorded from

Cameroon, DRC, Equatorial Guinea, Gabon and Nigeria; *P. uterobilateralis* from Cameroon, Gabon, Guinea, Liberia and Nigeria; *P. gondwaniensis* and *P. kerberti* from Cameroon; and *Paragonimus* sp. (where species was not specified) from Benin, Cote d'Ivoire, Gambia, Ghana, Libya, Republic of Congo, Senegal, South Africa and Tanzania, in addition to previously mentioned countries Cameroon, DRC, Equatorial Guinea, Gabon, and Nigeria.

Survey period: The survey includes all available publications since *Paragonimus*-like lung infections were first reported in Africa, from 1920 to the present. The number of studies by decade was as follows: 1910s (1), 1920s (1), 1930s (3), 1940s (3), 1950s (2), 1960s (8), 1970s (33), 1980s (27), 1990s (21), 2000s (16), and 2010s (26) (and an additional publication assessed data from 1970s-2000s). Publications increased substantially in the 1970s after the outbreak of paragonimiasis in Nigeria during the Biafran war, also known as the Nigerian Civil War (19; 1974; Fig 2). The majority of recent publications are from Nigeria (Fig 2). For *Poikilorchis congolensis*, apart from one relatively recent case report in the United Kingdom from a Nigerian patient (72), the remaining 6 publications dated from 1943 to 1975.

Study type: Literature by study type included: 30 case reports (sole human cases); 3 vet case reports (for sole domestic cat/dog cases); 11 case reviews (multiple cases, 3 also including surveys of crab intermediate hosts and 2 including clinical studies); 10 experimental studies; 8 clinical studies (including 3 drug treatment studies); 3 general biology studies, and 3 species descriptions. There were also 70 surveys in total: 27 solely human surveys (several also including clinical studies/drug treatment trials); 4 solely livestock surveys; 8 solely non-livestock mammal surveys; 3 solely snail surveys; 8 solely crab surveys; and the remainder of 20 various combinations of above survey categories. Experimental studies were carried out in the 1970s and 1980s (Fig 2), generally with Mandrills, *Mandrillus leucophaeus* or rhesus macaques, *Macaca mulatta* as the experimental host (73). An additional 3 surveys were questionnaire-based only, not included in further data analysis. For *P. congolensis*, apart from the species description (48), all publications were case reports or reviews (S1 Table).

Subset analysis

Prevalence and intensity of infection in humans: Prevalence estimates were included in 42 publications, of which 31 met our criteria for further analysis. In this subset, the study population size range was 127-3600 (average 1091). The number of studies by country was as follows: Nigeria (18), Cameroon (10), Liberia (1), Equatorial Guinea (1), and Gabon (1). Between 3-10 studies were carried out per decade since the 1970s (none from 1960s), and an additional survey was also published in the 1950s (18). The majority of studies were cross

sectional; both schoolchildren and adults were surveyed, and 7 surveyed school-aged populations only. In 10 studies other hosts were also surveyed e.g., crabs. Most studies surveyed populations within a given area, especially within known endemic regions, such as Cross River State in Nigeria, but many compared prevalence by locality within the surveyed region. Of the subset, 7 studies also screened for other parasites (soil transmitted helminths present for all, some studies additional helminths such as *Schistosoma mansoni* were found); and 6 also screened for TB (present in 3 studies).

The overall prevalence in the subset (*Paragonimus uterobilateralis* and *P. africanus* or unspecified) ranged between 0–27.0% (average: 8.4%). Average prevalence by decade, for both species combined: 1950s, 4% from 1 study only (**18**), 1960s no data, 1970s, 5.2% (5 studies), 1980s 8% (3 studies), 1990s 7.6% (8 studies), 2000s, 7.7% (6 studies), and 2010s, 12.3% (8 studies). In total 11 publications reported a study prevalence over 10%, 1 from Cameroon (74), and the remainder from Nigeria (22-24, 26, 33, 75-79).

Average prevalence across studies was significantly higher overall in *P. uterobilateralis*: at 12.4%, range 0-27% (1670/13,443, 12 studies), than *P. africanus*: at 2.9%, range 0.4-9.5% (263/8978, 8 studies, $P = 0.009$, Table 1). The average prevalence for surveys where the species was not specified (*Paragonimus* sp.) was 8.7%, range 0.2-20.8% (992/11415, 7 studies from Nigeria, 3 from Cameroon). Prevalence of *P. uterobilateralis* for studies in Nigeria only at 12.5% (11 studies), was significantly higher than for *P. africanus*, studies in Cameroon only at 3.4% (7 studies, $P = 0.008$).

For the intensity of infection subset (Table 1, S3 Table), 14 studies reported intensity in human surveys. The overall intensity of infection for *P. uterobilateralis* was 108.5 e/5g (Table 1, 9 studies, all in Nigeria apart from one in Liberia;80); for *Paragonimus* sp. where the species was not specified, 10.8 e/5g (4 studies). Range was recorded for 5 studies (Nigeria only), overall, 1-903 e/5g. No intensity of infection data for human surveys was available for *P. africanus*. In the three studies where both sputum (e/5g) and stool (epg) counts were reported, stool showed lower egg counts overall (23, 80, 81). In studies where intensity was assessed by category, 3 had standard categories (low at <50 e/5g, medium at 51-100 e/5g, and high at 101+ e/5g), where the overall averages were 43.3% for low intensity, 30.6% for medium and 25.9% for high intensity (33,75, 82).

Prevalence and intensity in other mammals: Mammal studies often involved opportunistic sampling and very small sample sizes, with few systematic surveys (but see 39, 55, 56, 83; S2 Table). Mammals surveyed included: primates (cercopithecids, lorids), Feliformia (viverrids,

herpestids); domestic animals (cats, dogs and pigs); Eulipotyphla (*Crocidura*) and Rodentia (*Atherurus* and assorted Muridae). The overall prevalences in all non-human mammal hosts were as follows: *P. africanus*, 43.2% (35/81, 5 studies), *P. uterobilateralis*, 35.7% (46/228, 4 studies), and *Paragonimus* sp., 6.5% (58/880, 7 studies). Incidences of infection were primarily evident in Feliformia and primates. Prevalences were as follows, in Feliformia: *P. africanus*, 58.8% (10/17, (*Civetticis civetta*, *Crossarchus obscurus*, *Nandinia binotata*, 4 studies), *P. uterobilateralis*, 82.5% (33/40, *Civetticis civetta*, *Crossarchus obscurus*, 3 studies); and in Primates *P. africanus*, 39.1% (25/64, *Cercocebus torquatus*, *Mandrillus leucophaeus*, *Perodicticus potto*, 3 studies), and *Paragonimus* sp., 14.4% (47/327, *Papio anubis*, 2 studies). Intensity was reported in three studies only, 96.7 epg of *P. africanus* in *Cercocebus torquatus* (39), 17 epg of *P. uterobilateralis* in *Civetticis civetta* (84), and *Paragonimus* sp. 47 epg in *Papio anubis* (55; Table 1).

Prevalence and intensity in crabs: Several systematic crab surveys undertaken near known foci of infection were reported in the literature. The overall prevalences for all genera/species pooled in freshwater crabs were as follows: *Paragonimus africanus*: range 0-88% (average 19.9%), (103/333 crabs surveyed total, 4 studies), *P. uterobilateralis*: range 0-100% (average 25.1%) (542/8300 crabs surveyed total, 14 studies). Prevalence and intensity of *Paragonimus* spp. varied by genera and species of crab (Table 1). For the most common crab host, *Sudanonautes africanus*, prevalence pooled across studies for *P. africanus* was significantly higher at 42.9% (103/240, 4 studies) than for *P. uterobilateralis* at 5.9% (P = 0.031, 156/2634, 5 studies). Overall intensity of infection in *S. africanus* across studies was also significantly higher for *P. africanus* at 54.6 metacercariae per host (106 crabs total, 2 studies) than *P. uterobilateralis* at 6 (P = 0.002, 34 crabs total, 2 studies; Table 1). *Sudanonautes aubryi* showed higher prevalence in *P. uterobilateralis* at 64.8% (57/88, 3 studies) than *P. africanus* at 6.3% (1/16, 1 study) but there were insufficient data to compare the difference (Table 1). Prevalence for *P. kerberti* in *S. africanus* was high at 32.9% (493 crabs in total, 1 study). In *S. pelli*, prevalence was low for both *P. africanus* at 1.5% (1/66, 1 study), and *P. uterobilateralis* at 3% (2/66, 1 study), although data were only available from 1 study which included co-infections (41). *Liberonautes* has been reported as a host only for *P. uterobilateralis* to date. *Liberonautes latidactylus* had both high prevalence of *P. uterobilateralis* (average 24.4%, 87/357, 4 studies) and intensity of infection (117.1, 85 crabs total, 3 studies, Table 1). Other species of *Liberonautes* where data were available showed low prevalence (and intensity) of infection, *L. chaperi* had a prevalence of 2.3% (1/4l, 1 study), and *L. nanoides*, 0.5% (4/753, 1 study; Table 1).

Egg size measurements: 37 studies included egg size measurements for *Paragonimus* spp., and 6 for *Poikilorchis congolensis* (Table 2, S4 Table). The overall averages and ranges for length and width were as follows (all hosts combined, weighted averages): *Paragonimus africanus*: length: average 91µm (+/-3.1 SD), range min and max averages, 76.4-107.5µm; width: average 49.5µm (+/-3.1 SD), range min and max averages, 43.6-59.1µm (from 2472 eggs, 9 studies); *Paragonimus uterobilateralis*: length: average 69.8µm (+/-4.1 SD), range min and max averages, 58.2-79.9µm; width: average, 41.1µm(+/-2.1 SD), range min and max averages, 36.2-46.1µm (from 3159 eggs, 9 studies); *Paragonimus* sp.: length: average: 88.2µm (+/-12.2SD), range min and max averages, 73.4-101.1µm; width: average, 52.7µm (+/-12 SD); range min and max averages, 43.1-60.1µm (from 472 eggs, 7 studies total, Table 2, Fig 3). For *Poikilorchis congolensis*: average length was 65µm (+/-7.4 SD), average width 43.3µm (+/-8.2 SD) (non-weighted average, Table 2); and range was 60-63× 40µm for all eggs apart from one measurement, at 60 × 80µm (S4 Table). Measurements were similar by host, i.e., eggs isolated from humans or other mammals showed similar values (Table 2). Metacercariae measurement sample sizes were very small with high standard deviations, but show in contrast to egg measurements, *P. uterobilateralis* metacercariae are generally larger sizes: *P. africanus* 564µm (+/-228.8 SD, from *Sudanonautes* spp., 3 studies), *P. uterobilateralis*, 701.6 (+/-119.4 SD, *Sudanonautes* and *Liberonautes*, 4 studies), and *Paragonimus* sp. (species not specified), 468.3 (+/-144.2 SD, *Sudanonautes*, *Liberonautes*, 2 studies, Table 2).

General qualitative observations were made for prevalence trends by demographics (age and sex) from the prevalence subset data, human surveys only; additionally observations were made on clinical symptoms, drug treatments and other diseases surveyed (where reported) in any publication. These observations are summarised in the following sections.

General epidemiology: Most studies in the human prevalence subset analysis examined prevalence differences by age (21 studies) and sex (19 studies). Highest prevalences were evident overall in younger age groups, where 10-20 year olds showed highest prevalence in 12 studies in total, including a longitudinal study which reported highest prevalences in this age category for three consecutive years (33); followed by 1-10 year olds in 7 studies. For the remaining 2 studies, one categorised broad age ranges, reporting highest prevalence in 15-44 year olds (77), and the other reported highest prevalence in the 40+ age category (24). Another study also reported second highest prevalence in 55-59 year olds (85). Apart from these studies, a sharp decline in prevalence was evident for older age groups. Often prevalence levels between age categories were similar in the younger age groups, e.g., 5-10-

and 11-20-year olds showing similar prevalences (22). Other surveys not in the subset noted a majority of cases in patients younger than 20 years (86). Examining differences in prevalence of infection by sex, 6 studies had higher prevalence in females than males, 10 studies, vice versa, and in 2, results were equivocal. Only 8 studies in total had significant differences, 3 with higher prevalence in females (all studies in Nigeria), and 5 with higher prevalence in males (4 studies in Nigeria, 1 in Cameroon). Unlike age, patterns of sex and prevalence of infection varied by study, and findings appeared to be study dependent, rather than indicating sex as a demographic factor affecting infection risk.

Clinical Findings: Clinical presentation of paragonimiasis was consistent across studies. Clinical signs and symptoms were mentioned in the majority of studies, and directly analysed or described in 18 publications, including 2 primate surveys. The recorded symptoms in order of most to least common were: cough, hemoptysis, and chest pain, and other symptoms including abdominal pain, headache, fever, dyspnoea, fatigue, uritcaria, eosinophilia, and diarrhoea (23, 28, 29, 33, 82, 87-92). Hemoptysis was very common, with 1 study also reporting that periods of egg excretion in sputum correlated with hemoptyis (93). Another study found no evidence of hemoptysis in any paragonimiasis case, although this was based on a small sample size (28). In terms of unusual symptoms, one study reported symptoms of epilepsy in paragonimiasis-positive patients (29) and four of suspected cases of cerebral paragonimiasis (symptoms of convulsions/hemiplegia or similar) (12, 18, 91, 92). Six studies also detailed radiological findings, such as pulmonary "shadows"; lesions and cavitations (87, 89, 92, 94, 93, 95). Two studies noted that it was difficult to distinguish from the radiological patterning of TB (87, 95), although the latter study noted evidence of subcutaneous tissue wasting in study participants with TB only.

Chemotherapies: Several publications studied effects of drug treatment, including the following chemotherapies: Niclofolan (96, 97); Tricloabendazole (98), Bithonol (96), Menichlopholan (99); and Praziquantel (71, 81, 100, 101), with the study population treated ranging from 6 to 360. Early treatments included emetine and chloroquine, and appeared to have had low efficacy, required repeat treatments and had significant side effects (11, 16, 18, 96). Later treatments included Bithonol, Menichlopholan and Niclofolan, with higher success rates, but still evidence of side effects (99, 96). Praziquantel was first trialled in 1980 (100) and found to be highly efficacious with few side-effects (71), and was used exclusively from the 1980s onwards for treatment (78, 87, 102).

Other diseases surveyed: Eight studies surveyed and reported on TB infections, 3 with study subjects selected from those positive for pulmonary symptoms, and 3 with randomly selected

study population (not showing symptoms). Two studies reported higher prevalence of TB than paragonimiasis, 19% vs 1.5% (103), and 16.9% vs 2.7% for (104). The remaining studies had higher prevalence for paragonimiasis than TB (27, 87, 88, 105), or only paragonimiasis and no TB infections were present (89, 90). In 2 studies, co-infections of TB and paragonimiasis were found, 1 case in Sachs et al. (106), and in Ibanga et al. (27) prevalences of TB only at 1.8%, and paragonimiasis at 9.4%, including 10 patients with co-infection (0.9% of total study population, 1100). There were also reports of patients previously treated for TB despite being tested as negative, and identified as positive for paragonimiasis in the given study (86). Ten human surveys and 2 mammal surveys also screened for other parasites, generally soil transmitted helminths. In these studies, *Ascaris lumbricoides*, *Tricuris tricura*, *Necator americana* and *Strongiloides stercoralis* (in decreasing order) were common; other parasites included *Schistosoma mansoni*, *Enterobium vermcularis*, *Entamoeba histolytica* and *Taenia saginata* (7, 28, 29, 55, 56, 74, 89, 90, 100, 107-109).

Mapping

For the mapping dataset, 132 publications were included (field surveys with viable occurrences/those including field-data), and for 120 publications at least 1 locality could be georeferenced (S5 Table). In total, 390 localities were selected for mapping (5 localities being *Poikilorchis congolensis* occurrences). Crab host genera show a wide distribution in West and Central Africa (*Sudanonautes* from Cote d'Ivoire to South Sudan, and *Liberonautes* from Senegal to Ghana, with occurrences spanning 1970-2017 (S Fig 1). The distribution of some of the freshwater crab host species (*Sudanonautes floweri*) extends beyond the known foci of paragonimiasis in the rainforests into the drier woodland and savannas of central and northern Nigeria, but only two outliers of *Paragonimus* have been reported from this region (Fig 4).

Examining distribution of *Paragonimus* occurrences by species: *P. africanus* was reported mainly in Cameroon, but also in Nigeria in 2 studies (39, 110), 1 occurrence from the Cross River Basin, the other apart further northwest at Enugu from a hospital patient, therefore low certainty (110). In addition, sole reports were evident for DRC (no supporting data; 111), Gabon (based on egg measurement and colour; 112) and Equatorial Guinea (based on egg measurement, morphology and colour; 113; Fig 5). *Paragonimus uterobilaterialis* in contrast was reported from Guinea to Gabon. Occurrences in outlier foci of KwaZulu-Natal in South Africa and Tanzania were reported as *Paragonimus* sp., although in Tanzania the

infections, in olive baboons, *Papio anubis* were suspected to be *Paragonimus uterobilateralis* based on egg morphology (55). Coordinate uncertainty was high for most localities, and those with low uncertainty occurrences from non-human hosts- crabs or wild-caught mammals, were all within the rainforest zone (Fig 5). There was evidence of temporal stability at endemic foci (S Fig 2).The main endemic regions of Southeast Nigeria and Southwest Cameroon show the close association of these foci with the Cross and Imo River systems in the River basin/Gulf of Guinea region (Fig 5).

Discussion

Infection in humans: We found numerous reports of significant recent transmission (1995-2016), with notably high prevalence and intensity in Southeast Nigeria in 15 studies, including 10 publications reporting prevalences over 10% (22-24, 26, 33, 75-79; Table 1). Recent transmission was also evident for Cote d'Ivoire (2006-2017; 40, 53, 114, 115), and Cameroon (1993-2014; 28, 29, 90, 91, 104). Recent high prevalence in Nigeria has been attributed to widespread eating of uncooked freshwater crabs due to a lack of both current knowledge and health campaigns on the risks (24, 116). In rural communities in Nigeria, ongoing poverty may contribute to dependence on crabs as a food source; and inadequate sanitation in endemic regions is likely also to increase local transmission (23). Prevalence in fact may be more widespread with under-reporting due to a lack of training of health workers to identify paragonimiasis (25, 117, 118); TB surveys omitting differential diagnoses for paragonimiasis (60, 119); and reliance on microscopy for diagnosis (where low intensity cases may go undetected) rather than the more sensitive serology methods (29, 119, 120). In contrast, serology is a ubiquitous diagnostic for *Paragonimus* in Asia (2). One relatively recent report showed evidence of co-infections of TB and paragonimiasis in Nigeria (27), supporting a case for under-reporting by misdiagnosis as TB as a distinct possibility. Overall, these prevalence figures are closely associated with endemic foci, and given the highly focal nature of the disease should not be extrapolated nationwide (1). Further, widespread praziquantel usage in many of the countries as chemotherapy for schistosomiasis may also be impacting paragonimiasis prevalence, but this has not been documented. However, the prevalence and intensity levels seen in these recent surveys in Africa are very troubling and would be cause for considerable attention in other less neglected tropical diseases (1).

Another unexpected result was the significantly higher overall prevalence of *P. uterobilateralis* at 12.4% than *P. africanus* at 2.9% ($P = 0.009$). For 11 surveys where the species identity was not reported, overall prevalence was 8.7%. As most of these studies took place in Nigeria, the majority were likely to be *P. uterobilateralis* (Fig 5), which fits the trend of higher prevalence of this species. This difference also reflects higher endemicity in Nigeria with overall prevalence of 12% (for *P. uterobilateralis*), vs *P. africanus* in Cameroon at 3.9%. Reasons for higher prevalence in *P. uterobilateralis* are unclear. *Paragonimus uterobilateralis* is much more broadly distributed, with a wider crab host range (Fig 4; S Fig 1), but *P. africanus* may be present in other foci, and there may be inaccuracies in species identification in some studies. Molecular analyses are required to ascertain true distribution.

In other mammals, prevalence was variable and sampling generally sporadic with very small sample sizes, but prevalence was higher than in humans. Infections were primarily reported in Feliformia (viverrids and herpestids); primates (cercopithedids and lorids), and less commonly in domestic animals (cats, dogs and pigs). In 3 systematic surveys in Primates, individuals were observed eating freshwater crabs (39, 55, 56), and in Bakuza (55), were identified as *Potamonautes* at Lake Tanganyika. Although the freshwater crabs in the latter study were not screened for infection, infected baboons showed evidence of advanced emaciation and high intensity of infection (121), suggesting significant zoonotic transmission in this region, although this requires investigation. The importance of paragonimiasis as a wildlife disease has not yet been fully realised. Further studies on the disease in mammals are needed using non-invasive faecal sampling (43).

Considering clinical findings and general epidemiology, there was a clear-cut association with younger age groups apart from two studies (24, 84), where the timing of onset of infection would fit with those infected as children during the Biafran war. Five studies reported epilepsy/convulsive episodes/hemiplegia in study participants positive for *Paragonimius* infection, suggesting the possibility of extrapulmonary cerebral localisation (12, 29, 91, 92), including one where 2 cases were assessed to be cerebral paragonimiasis (18). Extrapulmonary (and cerebral) paragonimiasis has been frequently reported in Asian paragonimiasis (1, 2), but appears to be rare in *Paragonimus* infections in Africa (45). Nevertheless, recent reports of high rates of epilepsy in endemic regions (85) and incidences in paragonimiasis positive patients (29) requires further study.

Infection in crabs: Both prevalence and intensity varied within genera according to *Paragonimus* species transmitted (Table 1). The most abundant host species *Sudanonautes africanus* had significantly higher prevalence (P = 0.031) and intensity (P = 0.002) of *P. africanus* than *P. uterobilateralis* infections overall. In *Liberonautes*, *L. latidactylis* showed high prevalence and intensity of *P. uterobilateralis* infections and in contrast *L. nanoides* very low levels of infection (122**;** Table 1). This presumably reflects susceptibility differences, which in turn would suggest crab species host range is a critical factor in *Paragonimus* distribution, although the key species have a wide range (Fig 4). Freshwater crabs are found throughout the range of *Paragonimus* in West and Central Africa, and it appears that the trematode is not host specific, developing in a number of different species depending on which are found in the focus of disease (10). The different species of *Paragonimus* may also adapt locally more to one host or another, resulting in local-scale differences in susceptibility. Data from co-infected *Sudanonautes africanus/pelli* in

Cameroon showed higher intensity of *P. africanus* within crabs in localities where it was more prevalent overall, likewise for *P. uterobilateralis* (41). It is not clear what is driving the predominance of either species of *Paragonimus* in this region where they overlap, potentially an interaction of factors at a local scale. It would be worthwhile to investigate these processes further in mixed infection foci.

Another notable finding in the literature is evidence of up to 100% prevalence and high intensity in crabs within endemic foci, e.g., the Lower Bakossi, Cameroon (41). This phenomenon has also been recorded at some foci for Asian *Paragonimus* (2). We hypothesise that evidence of highly concentrated prevalence and intensity in crabs in endemic foci suggests accumulation of the parasites in the crab over time. This would fit with a food-borne transmission pathway in the crabs, whereby the infected freshwater/semi-terrestrial snail host is eaten. Indeed, there is evidence that *Sudanonautes* in Nigeria feed on freshwater molluscs (123). Alternatively, cercariae may leave an infected snail, and either actively detecting and homing to a crab host, or adopting a sit-and-wait approach. Waterborne cercariae are a common life history strategy in trematodes (e.g., *Opisthorchis*), enabling wide dispersal. If a freshwater snail host, cercariae could disperse and detect freshwater crabs in waterbodies, or if semi-aquatic, by crawling on damp vegetation. The exact path of transmission at this point is not even known in the better studied Asian *Paragonimus* species (1).

The role of crabs in epidemiology overall is key, as metacercariae can persist in crabs for years, and intensity of infection can determine infectivity (4). There are however, few recent studies of *Paragonimus* infection in crabs (28, 60, 124), the last systematic studies being in the early 1990s (122, 125, 126, 127). Recent authors (28) have indicated that parasite intensity has declined in crab hosts, but there are insufficient recent data to compare with the surveys of Voelker & Sachs (41). Factors such as deforestation, hunting, pollution and waterway alterations are likely negatively impacting intermediate and definitive hosts of *Paragonimus*, which requires further investigation. Mammals in particular may have declined locally in areas under intensive hunting pressure (128).

Egg/metacercariae morphology and species distribution: Trends in egg size should be regarded with caution with potential misidentification given overlap in egg size morphology; that they can be affected by fixation/preparation method; and potentially by host (129). For the identification of African *Paragonimus* species however, egg (or metacercariae) morphology is generally the only method or data available. For Cote d'Ivoire, egg measurements of *Paragonimus* had a large size range (Fig 3, Table 2; S4 Table; 130). The variation may indicate the presence of both *P. africanus* and *P. uterobilateralis* in Cote

d'Ivoire. Further, metacercariae with morphology consistent with *P. africanus* (i.e., thick-walled cysts) are evident from studies where the metacercariae were isolated from *Liberonautes*/*Potamonautes* (see Figure 5 in 40). Both *Sudanonautes* and *Liberonautes* crab hosts are present in Cote d'Ivoire, the only country where they overlap and where *Paragonimus* has been found, apart from one livestock report from Ghana (83; Fig 4; S Fig 1). *Paragonimus africanus* has only previously been reported from Cameroon, Nigeria, DRC (111), Gabon (112) and Equatorial Guinea (Fig 5; 113). Potentially *P. africanus* is present in Cote d'Ivoire, but alternatively it may be an undescribed species (40, 131, 132). Other possibilities include a hybrid of *P. africanus* and *P. uterobilateralis*, or even a species complex (as may be the case for *P. westermani* in Asia; 2). Overall Cote d'Ivoire presents a complex disease landscape. *Paragonimus* infections have also been reported in the brackish-water crab *Callinectes* (53, 69, 114) but may be the same unidentified trematode in Traoré et al. (59; Figure 35 in 133**,** Figure 2 in 114). Clearly molecular studies are required to clarify *Paragonimus* taxonomy in this region (40).

Unlike *Paragonimus*, *Poikilorchis congolensis* egg size showed little variation, almost uniformly 60-63 × 40um (Table 2). Potentially it also has a crab intermediate host, and there is evidence of overlapping distribution with paragonimiasis (134), but very little is known of general biology. Oyediran et al. (49) observed that the characteristic retroauricular cysts where eggs are effectively trapped could represent an evolutionary dead-end for the parasite in humans, although Schuster et al. (72) also found evidence of eggs in stool. Work is needed to ascertain the definitive host. If there are many questions for *Paragonimus* in Africa, there are many more for *Poikilorchis.* With benign symptoms and limited public health impact underreporting may be high, particularly given the main endemic region appears to be DRC; but it does seem to be a rare parasite, although certainly worthy of separate investigation.

Distribution mapping: Mapping revealed few, but widespread outliers. Most occurrences were closely associated with endemic regions in rainforest zones (**135**) particularly low-uncertainty ones (Fig 5), illustrating the key importance of rainforest cover; but outliers also suggesting localised transmission where ecology is favourable, such as for cases in South Senegal and Gambia, although crabs have yet to be surveyed in this region. The main endemic foci are closely associated with the Imo and Cross River catchments (Fig 5). Although freshwater crab intermediate hosts are present in the Niger River catchment and Southwest Nigeria (Fig 4; S Fig 1), only 1 study has found infected crabs in these areas (136). Crab hosts are also present in northern Nigeria, but only one report of *Paragonimus* (in livestock) evident (137). Here limiting factors may be snail host distribution; or given that

these regions are outside of the main rainforest zone, mammal host abundances may be low. Endemic foci also show temporal stability (S1 Fig 2), similar to Asian *Paragonimus* species, e.g., at Sin Ho in Vietnam (2). Endemic zones essentially represent regions of overlapping host distribution, with suitable ecological conditions for all hosts.

Paragonimus uterobilateralis shows a more widespread distribution overall, *P. africanus* in contrast is geographically contained, within tightly clustered endemic foci (Fig 5). Liberia showed more dispersed occurrences, due potentially to its more extensive inland forest (135). Gaps were evident, with no records between Nigeria and Cote d'Ivoire, apart from Ghana (83) and a case in Benin (but potentially acquired via market crabs from Nigeria; 69). Likely ecological factors are in play with the markedly different ecology of the Dahomey gap in the region, with much drier conditions and forests fragmented with savannah (138). However, this could be partly due to sampling gaps, illustrated overall in the mapping (Figs 4, 5). DRC for example has the most extensive rainforest in Africa, but sparse occurrences have been recorded, spanning an immense area. Moving further east, evidence of *Paragonimus* in primates in Tanzania (55) suggests potential for contiguous distribution from the Central African forests at least to Lake Tanganyika. Additionally, there is a focus in KwaZulu-Natal, South Africa (44). Here potential freshwater crab hosts belong to *Potamonautes* (139), but no studies have screened these or any potential intermediate hosts for *Paragonimus* (Chris Appleton, pers. comm.). Little is known of the distribution of *Paragonimus* outside of the known endemic regions; *Paragonimus* spp. could be distributed far more widely than the known foci in West and Central Africa.

First intermediate host: One of the more intriguing aspects of paragonimiasis in Africa is that the first intermediate snail host is yet to be identified (43). Studies investigating snail hosts so far include the following as potential species: *Afropomus balanoidea* (34), *Homorus* (*Striosubulina*) *striateila* (35, 36), *Potadoma freethii* (26, 31, 32), *Potadoma sanctipauli* (34), Achatinidae (30), and *Achatina achatina* (Moyou-Somo pers. comm.). Some of these studies may have actually identified microcercous cercariae of *Paragonimus* in molluscs (30, 35) but morphological and molecular data in combination are required for confirmation. As Asian *Paragonimus* has a wide host range within Rissoidea and Cerithidoidea (9), African species may also infect a range of snails, contrary to the suggestion of Cumberlidge et al. (43) of a specific snail host range. The authors also proposed the first intermediate host snail may be amphibious/semi-terrestrial rather than fully aquatic, inhabiting the moist conditions of the tropical forest floor and picking up miracidia from a scat, in turn consumed by a semi-terrestrial freshwater crab, thereby infecting the second intermediate host. Transmission may

even occur in both freshwater and semi-terrestrial snail species, especially given a likely wide snail host range.

Limitations of study; risk of bias and systematic review challenges: Unfortunately, conflict and political instability has impacted sampling. Paragonimiasis is most likely present in Sierra Leone, although to date no surveys have been carried out to our knowledge; no recent surveys have been carried out in Liberia; and no surveys done at all in the DRC, all occurrences obtained from historic case reports only. Overall, reported prevalences are potentially underestimates, partly because they are based on microscopy only which lacks sensitivity. Improved diagnostics will likely lead to evidence of greater numbers of cases. However, the high prevalences reported in recent surveys also reflects a sampling bias toward high endemicity areas. Bias within studies includes possibility of species misidentification; therefore higher prevalence of *P. uterobilateralis* would need to be confirmed with longitudinal molecular studies. Intensity in crabs is complicated by age (size) of crabs, with studies showing higher intensity in larger crabs (41, 110, 140), but there were insufficient individual measurements for further analysis. Another bias is non-recording of study period in many cases, and temporal inaccuracy when infections were acquired when onset of symptoms was not clear in case reports, although this was mitigated by assessing time period by decade only. Some of the contributions included here were challenging to obtain, and undoubtedly some publications with medical implications have remained overlooked until now. This current search did not include national Ministries of Health records, potentially useful particularly for countries where paragonimiasis surveys have not been published, like DRC. Overall however, the risk of bias at the study selection stage was limited, our search included all languages; and we present a near-comprehensive literature review of all publications to date on African *Paragonimus* that considerably expands and updates the recent study by Cumberlidge et al. (43).

Conclusions and future directions

One hundred and forty-two papers were reviewed, where significant ongoing transmission of paragonimiasis with high prevalence and intensity of infection was uncovered, particularly for Southeast Nigeria. Across studies, we found higher prevalence overall for *P. uterobilateralis* than *P. africanus* in human surveys; variable prevalence and intensity of *Paragonimus* spp. infection in different crab hosts; and the potential endemicity of *P. africanus* in Cote d'Ivoire. In human surveys, the majority of the disease burden was consistently higher in younger age groups (<20 years). This review also suggests paragonimiasis is a wildlife disease of importance in primates and Feliformia (viverrids and herpestids) in Africa. Mapping showed the close association of *Paragonimus* distribution with rainforest zones, but also outliers indicating localised transmission at locations with suitable ecology for all hosts. Temporal stability in endemic foci was evident. The mapping also illustrates extensive data gaps and under-sampling, and more widespread transmission than previously reported in Cumberlidge et al. (43). Our systematic review has greatly improved the existing body of knowledge on paragonimiasis in West and Central Africa. In terms of future work, molecular studies are urgently required to address major knowledge gaps in general biology. Metabarcoding of infected crabs could provide an approach to investigate potential first intermediate hosts via identification of diet items of the intermediate host crab species, a potential transmission pathway. Metabarcoding and eDNA techniques could also help narrow the field of potential first intermediate hosts, which could then be intensively examined with dissection as prevalences may be low in the snail host, as seen in schistosomiasis (141). Bayesian spatial modelling could illuminate potential distribution outside key endemic zones. Extensive human surveys in endemic foci, aided by better diagnostics, are needed to establish the true extent of human infection, and treatment and control need greater consideration. As paragonimiasis is a food borne disease, much could be done to promote safer eating practices. In the wake of the COVID-19 crisis, there is a renewed focus on zoonotic transmission and the One Health approach. Paragonimiasis-endemic regions, such as Cameroon, Cote d'Ivoire and Nigeria have a public health landscape of risk of infection with multiple NTDs as well as infectious diseases like malaria, TB, and others. To achieve goals on reducing transmission of the NTDs, particularly in a One Health context, there is a need to understand all these diseases collectively. This is a call to action for international NTD community, to commit programmes of research into paragonimiasis in Africa.

Acknowledgements

The authors are immensely grateful to the following people for their assistance: Rosie Jones, John Rose, Angela Thresher, Ben Nathan, Helen Pethers and Nial Briggs at the NHM library for assistance with interlibrary loans and scanning maps; authors David Aka, David Blair and Chris Appleton for sending papers; Chiho Ikebe for translation of Yamamoto et al. (1979), and authors Agathe Nkouawa, Richard Bradbury, Fabrice Zobel Cheuyem Lekeumo, Basil Okeahialam, and Roger Moyou-Somo for sending additional data from published works.

Funding: No funding was directly received for this work. MR is generally supported by Wellcome Trust SCAN project [104958/Z/14/Z].

Author contributions:

MER and JW read the literature, extracted relevant data and undertook exploratory mapping, JW translated German texts, MER undertook data analysis, wrote the manuscript and produced maps and figures, NC, PC, DR, AE and JW provided edits and comments on the manuscript. All authors read and approved the final draft.

References

1. Blair D. Paragonimiasis. In: Toledo R, Fried B. (eds.) Digenetic Trematodes. Advances in Experimental Medicine and Biology. Springer Nature Switzerland AG, Cham, Switzerland, 2019; 1154:105–138. https://doi.org/10.1007/978-3-030-18616-6_5

2. Yoshida A, Doanh PN, Maruyama H. Paragonimus and paragonimiasis in Asia: An update. Acta tropica. 2019 Nov 1;199:105074. https://doi.org/10.1016/j.actatropica.2019.105074

3. WHO. Control of foodborne trematode infections. Report of a WHO study group. World Health Organ. Technical Report Series. 1995; 849:1–157. PMID: 7740791

4. Yokogawa S, Cort WW, Yokogawa M. Paragonimus and paragonimiasis. Experimental Parasitology. 1960; 10(2):139-205.

5. Sachs R, Cumberlidge N. The dog as natural reservoir host for Paragonimus uterobilateralis in Liberia, West Africa. Annals of Tropical Medicine and Parasitology. 1990a; 84(1):101–102.

6. Voelker J, Sachs R, Volkmer KJ, Braband H. Zur Epidemiologie der Paragonimiaais bei Mensch und Tier in Nigeria, Westafrika. Veterinär-medizinische Nachrichten. 1975; 1/2:158–172.

7. Vogel H, Crewe W. Beobachtungen über die Lungenegel-Infektion in Kamerun (Westafrika). Zeitschrift für Tropenmedizin und Parasitologie. 1965; 16(2):109–125. PMID: 5851297

8. Voelker J, Vogel H. Zwei neue Paragonimus-Arten aus West-Africa: *Paragonimus africanus* und *Paragonimus uterobilateralis* (Troglotrematidae, Trematoda). Zeitschrift für Tropenmedizin und Parasitologie. 1965; 16:125–147.

9. Blair D, Davis GM, Wu B. Evolutionary relationships between trematodes and snails emphasizing schistosomes and paragonimids. Parasitology. 2001; 123(7):229–243. https://doi.org/10.1017/S003118200100837x PMID: 11769286

10. Cumberlidge N. The freshwater crabs of West Africa: family Potamonautidae. IRD editions; 1999.

11. Nwokolo C. Paragonimiasis in Eastern Africa. Journal of Tropical Medicine and Hygiene. 1964; 67(1):1–4.

12. Onorato, R. La paragonimiasi in Tripolitania. Archivio Italiano di Scienze Mediche Coloniali 1920; 1:1-13

13. Mönnig, H. O. Check list of the worm parasites of domesticated animals in South Africa. Pretoria: Government Printer and Stationery Office. 13th and 14th Reports of the Director of Veterinary Education and Research, 1928; 2:801–840.

14. Libert CEMJ. A Case of Paragonimiasis. West African Medical Journal. 1932; 5(3):51.

15. van Hoof L. Case of *Paragonimus* infection in Belgian Congo. Annales de la Société Belge de Médecine Tropicale. 1933; 13:473–478.

16. Bertrand, J. Distomatose pulmonaire ou hémoptysie pulmonaire à Paragonimus. Annales de la Société Belge de Médecine Tropicale. 1947; 27:1-4.

17. Cannon, D. A. Eggs of Paragonimas in sputum of patient in the Cameroons. Transactions of the Royal Society of Tropical Medicine and Hygiene. 1949; 42(4):322

18. Zahra A. Paragonimiasis in the Southern Cameroons: a Preliminary Report. West African Medical Journal. 1952; 1(2):75–82.

19. Nwokolo C. Endemic paragonimiasis in Africa. Bulletin of the World Health Organization. 1974; 50(6):569–571. PMID: 4549201

20. Nwokolo C. Outbreak of paragonimiasis in Eastern Nigeria. The Lancet. 1972a; 299(7740):32–33. https://doi.org/10.1016/S0140-6736(72)90017-7 PMID: 4108826

21. Nwokolo C. Endemic paragonimiasis in Eastern Nigeria. Clinical features and epidemiology of the recent outbreak following the Nigerian civil war. Tropical and Geographical Medicine. 1972b; 24(2):138–47. PMID: 5037686

22. Iboh CI. Investigation of Crab-eating Influence on Paragonimiasis Infection in Six Communities of Abayong from Cross River State Nigeria. South Asian Journal of Parasitology. 2018; 1(1):1–9. https://doi.org/10.9734/SAJP/2018/41742

23. Eze JC. Epidemiological Pattern of Presentation of Paragonimus Infection in the Human Host in South East Nigeria and Their Correlative Sonographic Findings, 2015; University of Nigeria, Thesis.

24. Uttah EC, Etim SE, Ibe DC. Familial and occupational clustering of paragonimiasis in a riverine community in Eastern Nigeria. Transnational Journal of Science and Technology. 2013; 3(1):25–35.

25. Eke RA, Udochi I, Nwosu M, Enwereji EE, Emerole CV. Paragonimiasis Reemergence in Nigeria: Predisposing Factors and Recommendations for Early Intervention and Everlasting Eradication. ISRN Infectious Diseases. 2013; 2013(257810): 4 pages. http://dx.doi.org/10.5402/2013/257810

26. Asor JE, Ibanga ES, Arene FI. The Epidemiology of Pulmonary Paragonimiasis in Cross River Basin in Nigeria: Update on infection prevalence and distribution of the snail and crab intermediate hosts. Mary Slessor Journal of Medicine. 2003a; 3(1):5–12. https://doi.org/10.4314/msjm.v3i1.10988

27. Ibanga, E. S., Arene, F. O. I., & Asor, J. E. Association of pulmonary paragonimiasis with active pulmonary tuberculosis in rural Yakurr community in Cross River Basin, Nigeria. Mary Slessor Journal of Medicine. 2003; 3(1):19-23.

28. Moyou-Somo R, Mfouapong-Ewane HB, Nkoa T, Etaluka-Mungo B, Kum-Kan W, Kefie-Arrey C. A new focus of pleuro-pulmonary paragonimiasis in Manjo Health District, Littoral Region of Cameroon. International Journal of Tropical Disease and Health. 2014; 4(9):963–972. https://doi.org/10.9734/IJTDH/2014/11252

29. Nkouawa A, Okamoto M, Mabou AK, Eding E, Yamasaki H, Sako Y, Nakao M, Nakaya K, Blair D, Agatsuma T, Enyong P, Shibahara T, Moyou-Somo R, Ito A. Paragonimiasis in Cameroon: molecular identification, serodiagnosis and clinical manifestations. Transactions of the Royal Society of Tropical Medicine and Hygiene. 2009; 103(3):255–261. https://doi.org/10.1016/j.trstmh.2008.09.014 PMID: 18992906

30. Moyou-Somo R, Guemgne-Simo G. Paragonimiasis in southwest Cameroon: isolation of microcercous cercariae from land snails caught in a *Paragonimus africanus* endemic zone. Wilderness and Environmental Medicine. 1995; 6(1):44–47. https://doi.org/10.1016/S1080-6032(13)80008-7

31. Moyou-Somo R, Enyong PA, Kouamouo J. Isolation of *Paragonimus africanus* cercariae from a wild caught mollusc Potadoma freethi. XI Intentional Congress of Tropical Medicine and Malaria, Canada: Calgary. 1984; pp. 208.

32. Arene FO, Ibanga ES, Asor JE. Freshwater snail and crab intermediate hosts of *Paragnimus* species in two rural communities in Cross River Basin Nigeria. Global Journal of Pure and Applied Sciences. 1999; 5:184–188.

33. Udonsi JK. Endemic *Paragonimus* infection in the Upper Igwun Basin, Nigeria: a preliminary report on a renewed outbreak. Annals of Tropical Medicine and Parasitology. 1987; 81(1):57–62. https://doi.org/10.1080/00034983.1987.11812091 PMID: 3675045

34. Voelker, J. Morphologisch-taxonomische Untersuchungen über Paragonimus uterobilateralis (Trematoda, Troglotrematidae) sowie Beobachtungen über den Lebenszyklus und die Verbreitung des Parasiten in Liberia.Tropenmedizin und Parasitologie. 1973; 24:4–20

35. Sachs R, Cumberlidge N. Isolation of microcercous cercariae from land snails caught in an endemic focus of *P. uterobilateralis* in Liberia, West Africa. Tropenmedizin und Parasitologie. 1989; 40(1):69–72. PMID: 2740730

36. Sachs R, Cumberlidge N. Notes on the ecology of *Homorus striatellus* (Rang, 1831), snail host of microcercous cercariae in Liberia, West Africa. Zeitschrift für Angewandte Zoologie. 1991a;78(1):45–53.

37. Bayssade-Dufour C, Chermette R, Šundić D, Radujković BM. *Paragonimus gondwanensis* n. sp. (Digenea, Paragonimidae), parasite of mammals (humans and carnivores) in Cameroon. Ecologica Montenegrina. 2014; 1(4):256–267. https://doi.org/10.37828/em.2014.1.34

38. Bayssade-Dufour C, Chermette R, Šundić D, Radujković BM. *Paragonimus kerberti* n.sp. (Digenea, Paragonimidae), parasite of carnivores in Cameroon. Ecologica Montenegrina. 2015; 2(3):71–277.

39. Friant S, Brown K, Saari MT, Segel NH, Slezak J, Goldberg TL. Lung fluke (*Paragonimus africanus*) infects Nigerian red-capped mangabeys and causes respiratory disease. International Journal for Parasitology: Parasites and Wildlife. 2015; 4(3):329–332. https://doi.org/10.1016/j.ijppaw.2015.08.003 PMID: 26543803

40. Aka NA, Sanogho Y, Yapo-Kouadio CG, Dou GSP, Zika KD, Adoubryn KD. Persistance du foyer de distomatose pulmonaire a Lakota (Cote d'Ivoire): cas du village de Gbahiri. Bio-Africa Revue. 2018; 17):54–59.

41. Voelker J, Sachs R. Über die Verbreitung von Lungenegeln, *Paragonimus africanus* und *P. uterobilateralis,* in WestKamerun und OstNigeria auf Grund von Untersuchungen an Süsswasserkrabben auf Befall mit Metacercarien. Tropenmedezin und Parasitologie. 1977a; 28(1):29–133. PMID: 871036

42. Kittur N, King CH, Campbell Jr CH, Kinung'hi S, Mwinzi PN, Karanja DM, N'Goran EK, Phillips AE, Gazzinelli-Guimaraes PH, Olsen A, Magnussen P. Persistent hotspots in schistosomiasis consortium for operational Research and evaluation studies for gaining and sustaining control of schistosomiasis after four years of mass drug administration of praziquantel. American Journal of Tropical Medicine and Hygiene. 2019; 101(3):617–627. https://doi.org/10.4269/ajtmh.19-0193 PMID: 31287046

43. Cumberlidge N, Rollinson D, Vercruysse J, Tchuem Tchuenté L-A, Webster B, Clark PF. *Paragonimus* and paragonimiasis in West and Central Africa: unresolved questions.

Parasitology. 2018; 145(13):1749–1757. https://doi.org/10.1017/S0031182018001439 PMID: 30210013

44. Appleton CC. Paragonimiasis in KwaZulu-Natal province, South Africa. Journal of Helminthology. 2014; 88(1):123–128. https://doi.org/10.1017/S0022149X1200083 PMID: 23253517

45. Mazigo HD, Morona D, Kweka EJ, Waihenya R, Mnyone L, Heukelbach J. Epilepsy and tropical parasitic infections in Sub-Saharan Africa: a review. Tanzania Journal of Health Research. 2013; 15(2): 102–19. https://doi.org/10.4314/thrb.v15i2.5 PMID: 26591716

46. Aka NA, Adoubryn K, Rondelaud D, Dreyfuss G. Human paragonimiasis in Africa. Annals of African Medicine. 2008a; 7(4):153–162. https://doi.org/10.4103/1596-3519.55660 PMID: 19623916

47. Moher D, Liberati A, Tetzlaff J, Altman DG. Preferred Reporting Items for Systematic Reviews and Meta-Analyses: The PRISMA Statement. PLoS Medicine. 2009; 6(7):e1000097. https://doi.org/10.1371/journal.pmed.1000097 PMID: 19621072

48. Fain A, Vandepitte J. A new trematode: *Poikilorchis congolensis*, n.g., n.s., living in subcutaneous retrouricular cysts in man from the Belgian Congo. Nature. 1957; 179:740. http://dx.doi.org/10.1038/179740a0 PMID: 13418777

49. Oyediran ABOO, Fajemisin AA, Abioye AA, Lagundoye SB, Olugbile AOB. Infection of the mastoid bone with a *Paragonimus*-like trematode. American journal of tropical medicine and hygiene. 1975; 24(2):268–273. https://doi.org/10.4269/ajtmh.1975.24.268 PMID: 1119667

50. van Nieuwenhuyse E, Gatti F. A case of chronic mastoid infestation by a rare parasite (*Poikilorchis congolensis*). Acta OtoLaryngologica. 1968; 66(5):444–448. https://doi.org/10.3109/00016486809126309 PMID: 5732650

51. Pozio E. Current status of food-borne parasitic zoonoses in Mediterranean and African regions. Southeast Asian Journal of Tropical and Medical Public Health. 1991; 2285–87. PMID: 1822944

52. Goldsmid JM. Ecological and cultural aspects of human trematodiasis (excluding schistosomiasis) in Africa. The Central African Journal of Medicine. 1975; 21(3):49–53. PMID: 804995

53. Aka NA, Assoumou A, Adoubryn KD., Domoua K, Kouadio F, Moyou-Somo R, Nakamura-Uchiyama F, Nawa Y, Rondelaud D, Dreyfuss G. documents sur la séroépidémiologie de la paragonimose humaine au centre anti-tuberculeux de Divo, République de Côte d'Ivoire (Afrique de l'Ouest). Parasite. 2008c; 15(2):157–161. https://doi.org/10.1051/parasite/2008152157 PMID: 18642509

54. Malvy D, Ezzedine KH, Receveur MC, Pistone T, Mercie P, Longy-Boursier M. Extra-pulmonary paragonimiasis with unusual arthritis and cutaneous features among a tourist

returning from Gabon. Travel Medicine and Infectious Disease. 2006; 4(6):340–342. https://doi.org/10.1016/j.tmaid.2006.01.003 PMID: 17098631

55. Bakuza JS. Epidemiology of Schistosoma mansoni infection in sympatric humans and non-human primates in the Gombe ecosystem Tanzania. 2012; University of Glasgow. PhD thesis.

56. Müller-Graf CDM, Collins DA, Woolhouse MEJ. Intestinal parasite burden in five troops of olive baboons (*Papio cynocephalus anubis*) in Gombe Stream National Park, Tanzania. Parasitology. 1996; 112(5): 489–497. https://doi.org/10.1017/S0031182000076952 PMID: 8677138

57. Plouvier S, Loubière R, Colette J, Prost A, Baudoin C, Sellin B. Un cas de distomatose en Haute-Volta. In: Conférence technique de l'OCCGE. Bobo-Dioulasso: OCCGE, multigr. Conférence Technique de l'OCCGE, 15., Bobo-Dioulasso (BF), 1975/04/07-11. 1975; 7 p. http://www.documentation.ird.fr/hor/fdi:08573

58. Wickham H, Averick M, Bryan J, Chang W, McGowan LD, François R, Grolemund G, Hayes A, Henry L, Hester J, Kuhn M. Welcome to the Tidyverse. Journal of Open Source Software. 2019;4(43):1686. https://doi.org/10.21105/joss.01686

59. Traoré SG, Koussemon M, Odermatt P, Aka ND, Adoubryn KD, Assoumou A, Dreyfuss G, Bonfoh B. Risque de contraction de trématodoses alimentaires avec la consommation des crustacés vendus sur les marchés d'Abidjan. Revue Africaine de Santé et de Productions Animales. 2010; 120:514–520.

60. Uttah EC. Prevalence of human edible crabs infected with *Paragonimus Uterobilateralis* Metacercariae in southeastern Nigeria. Pacific Journal of Medical Sciences. 2013; 11(1):12–20.

61. Hijmans RJ, Guarino L, Cruz M, Rojas E. Computer tools for spatial analysis of plant genetic resources data: 1. DIVA-GIS. Plant Genetic Resources Newsletter. 2001; 127:15–19.

62. Sachs R, Distribution of Paragonimus uterobilateralis metacercariae in Liberonautes latidactylus, Annual Report 1985, Liberia Research Unit of the Tropical Institute Hamburg 1985c:4–47.

63. Chukwu O, Degiri A, Uzoigwe RN, Chukwe ID, Ogo IN, Ojemudia IT, Maguga LJ, Nsue M, Olabode OA. Polyparasitic infections in school children of Equatorial Guinea. International Journal of Natural and Applied Sciences. 2009; 5(1). https://doi.org/10.4314/ijonas.v5i1.49948

64. N'Dzossi AAC. Observations clinique et épidémiologique du premier cas de paragonimose humaine au Congo-Brazzaville (Doctoral dissertation). University of Bordeaux. 2000.

65. MA, Egbuna WO. Paragonimiasis in Lagos: a report of two cases. Nigerian Medical Journal. 1979; 9(5–6):641–644. PMID: 525059

66. Bosse, D. Endémicité et parasitoses autochtones en Afrique noire: à propos d'un foyer insolite de distomatose pulmonaire en Côte d'Ivoire. (Doctoral dissertation). University of Montpellier, 1984, 112 p.

67. Sam-Abbenyi A. Paragonimose endemique dans le Lower Mundani, arrondissement de Fontem, une etude preliminaire. Bulletin de liaison et de documentation de l'OCEAC. 1982; 52:42–44.

68. Morter R, Adetifa I, Antonio M, Touray F, De Jong BC, Gower CM, Gehre F. Examining human paragonimiasis as a differential diagnosis to tuberculosis in the Gambia. BMC Research Notes. 2018; 11:31. https://doi.org/10.1186/s13104-018-3134-y PMID: 29334998

69. Aka NA, Allabi AC, Dreyfuss G, Kinde-Gazard D, Tawo L, Rondelaud D, Bouteille B, Avodé G, Anagonou SY, Gninafon M, Massougbodji A, Dumas M. Observations épidémiologiques sur le premier cas de paragonimose humaine et les hôtes intermédiaires potentiels de Paragonimus sp. au Bénin. Bulletin de la Société de Pathologie Exotique et de ses filiales. 1999; 92(3):191–194. PMID: 10472447

70. Traoré SG, Odermatt P, Bonfoh B, Utzinger J, Adoubryn KD, Assoumou A, Dreyfuss G, Koussémon M. No Paragonimus in high-risk groups in Côte d'Ivoire, but considerable prevalence of helminths and intestinal protozoon infections. Parasites & Vectors. 2011;4(1):96.

71. Sam-Abbenyi A. Endemic pulmonary paragonimiasis in Lower Mundani (Fontem district of southwest Cameroon). Results of treatment with praziquantel. Bulletin de la Société de Pathologie Exotique et de ses filiales. 1985; 78(3):334–341. PMID: 4028318

72. Schuster H, Agada FO, Anderson AR, Jackson RS, Blair D, McGann H, Kelly G. Otitis media and a neck lump - Current diagnostic challenges for *Paragonimus*-like trematode infections. Journal of Infection. 2007; 54(2):e103–6. https://doi.org/10.1016/j.jinf.2006.05.011 PMID: 16824610

73.V oelker J, Sachs R. Affen als natürliche und experimentelle Endwirte afrikanischer Lungenegel (*Paragonimus africanus* und *P. uterobilateralis*) Tropenmedizin und Parasitologie. 1977; 28:137–144. PMID: 407679

74. Timsit, D. La paragonimose pulmonaire au Cameroun. Médecine Tropicale. 1978; 38:287–297.

75. Uttah EC. Paragonimiasis and Renewed Crab-Eating Behavior in Six Communities from Two Ethnocultural Clusters in Southeastern Nigeria. International Scholarly Research Notices, 2012; 2013:5 p. https://doi.org/10.5402/2013/569485

76. Okoro N, Onyeagba RA, Anyim C, Eda OE, Okoli CS, Orji I, Okonkwo EC, Ekuma UO, Agah MV. Prevalence of *Paragonimus* Infection. American Journal of Infectious Diseases. 2013; 9(1):17–23. https://doi.org/10.3844/ajidsp.2013.17.23

77. Abraham JT, Akpan PA. Current status of paragonimiasis in Old Netim 1 Akamkpa Local Government Area of Cross River State-Nigeria. Global Journal of Medical Sciences. 2009; 8(1, 2):17–22.

78. Asor JE, Ibanga ES, Arene FOI. *Paragonimus uterobilateralis*: peak period of egg output in sputum of infected subjects in Cross River Basin, Nigeria. Mary Slessor Journal of Medicine. 2003b; 3(1):24–27. https://doi.org/10.4314/msjm.v3i1.10991

79. Arene FO, Ibanga E, Asor JE. Epidemiology of paragonimiasis in Cross River basin, Nigeria: prevalence and intensity of infection due to *Paragonimus uterobilateralis* in Yakurr local government area. Public Health. 1998; 112(2):119–122. https://doi.org/10.1038/sj.ph.1900382 PMID: 9581454

80. Sachs R, Albiez EJ, Voelker J. Prevalence of *Paragonimus uterobilateralis* infection in children in a Liberian village. Transactions of the Royal Society of Tropical Medicine and Hygiene. 1986; 80:800–801. https://doi.org/10.1016/0035-9203(86)90387-1 PMID: 3603619

81. Udonsi JK. Clinical field trials of praziquantel in pulmonary paragonimiasis due to *Paragonimus uterobilateralis* in endemic populations of the Igwun Basin, Nigeria. Tropical Medicine and Parasitology. 1989; 40(1):65–68. PMID: 2662354

82. Ibanga ES, Eyo VM. Pulmonary paragonimiasis in Oban community in Akamkpa Local Government area, Cross River State, Nigeria: prevalence and intensity of infection. Transactions of the Royal Society of Tropical Medicine and Hygiene. 2001; 95(2):159–160. https://doi.org/10.1016/S0035-9203(01)90144-0 PMID: 11355547

83. Permin A, Yelifari L, Bloch P, Steenhard N, Hansen NP, Nansen P. Parasites in cross-bred pigs in the Upper East Region of Ghana. Veterinary Parasitology. 1999; 87(1):63–71. https://doi.org/10.1016/s0304-4017(99)00159-4 PMID: 10628701

84. Sachs R, Voelker J. The African civet as a natural host of the lung flukes observed in Liberia. Annual Report, Liberia Research Unit of the Tropical Institute Hamburg. 1985d:p 48.

85. Wokem G. Preliminary Survey of Epidermiological and Socio-Economic Studies on pulmonary paragonimiasis in part of Imo Rivers basin, Imo state, Nigeria on Pulmonary Paragonimiasis in part of Imo Rivers Basin, Imo State, Nigeria. Niger Delta Biologica. 2004; 4(2):82–87.

86. Ogakwu M, Nwokolo C. Radiological findings in pulmonary paragonimiasis as seen in Nigeria: a review based on one hundred cases. The British Journal of Radiology. 1973; 46(549):699-705. https://doi.org/10.1259/0007-1285-46-549-699

87. Oloyede IP, Ekanem EE, Nyong EE. Prevalence, co-prevalence and risk factors of pulmonary paragonimiasis and pulmonary tuberculosis in Nigerian Children in the Niger Delta Area. East African Medical Journal. 2013; 90(6):182–188. PMID: 26859024

88. Umoh NO, Useh MF. Epidemiology of paragonimiasis in Oban community of Cross River State, Nigeria. Mary Slessor Journal of Medicine. 2009; 9(1):1-10 https://doi.org/10.4314/msjm.v9i1.46637

89. Moyou-Somo R, Tagni-Zukam D. Paragonimiasis in Cameroon: clinicoradiologic features and treatment outcome. Médecine tropicale: revue du Corps de santé colonial. 2003; 63(2):163–7. PMID: 12910655

90. Moyou-Somo R, Kefie-Arreym C, Dreyfuss G, Dumas M. An epidemiological study of pleuropulmonary paragonimiasis among pupils in the peri-urban zone of Kumba town, Meme Division, Cameroon. BMC Public Health. 2003; 3:40. https://doi.org/10.1186/1471-2458-3-40 PMID: 14678562

91. Ollivier G, Boussinesq M, Albare JJ, Cumberlidge N, Farhati K, Chippaux JP, Bayssade-Dufour C. Étude épidémlologique d'une distomatose à *Paragonimus* sp. au Sud-Cameroun. Bulletin de la Société de Pathologie Exotique et de ses filiales. 1995; 88(4):164–169. PMID: 8640078

92. Kum PN, Nchinda TC. Pulmonary paragonimiasis in Cameroon. Transactions of the Royal Society of Tropical Medicine and Hygiene. 1982;76(6):768–772. https://doi.org/10.1016/0035-9203(82)90102-X PMID: 7164143

93. Volkmer KJ. Diagnostic pattern of African paragonimiasis. Annales de la Société belge de médecine tropicale. 1975; 55(5):535–453.

94. Volkmer KJ, Braband H. Das Röntgenbild der afrikanischen Paragonimiasis. RöFo-Fortschritte auf dem Gebiet der Röntgenstrahlen und der bildgebenden Verfahren. 1975; 122(3);265–267.

95. Ogakwu M, Nwokolo C. Radiological findings in pulmonary paragonimiasis as seen in Nigeria: a review based on one hundred cases. The British Journal of Radiology. 1973; 46(549):699–705.

96. Nwokolo C. Treatment of African paragonimiasis. Tropenmedizin und Parasitologie. 1976; 27:53–55.

97. Ripert C, Carrie J, AmbroiseThomas P, Baecher R, Kum NP, SameEkobo A. Étude épidémiologique et clinique de la paragonimose au Cameroun. Résultats du traitement par le niclofolan. Bulletin de la Société de Pathologie Exotique et de ses filiales. 1981; 74(3):319–331. PMID: 7285268

98. Ripert C., Couprie B, Moyou R, Gaillard F, Appriou M, Tribouley-Duret J. Therapeutic effect of triclabendazole in patients with paragonimiasis in Cameroon: a pilot study. Transactions of the Royal Society of Tropical Medicine and Hygiene. 1992; 86(4):417–417. https://doi.org/10.1016/0035-9203(92)90247-a PMID: 1440821

99. Nwokolo C, Volkmer KJ. Single dose therapy of Paragonimiasis with menichlopholan. American Journal of Tropical Medicine and Hygiene. 1977; 26:4. https://doi.org/10.4269/ajtmh.1977.26.688 PMID: 889010

100. Monson MH, Koenig JW, Sachs A. Successful treatment with praziquantel of six patients infected with the African lung fluke, *Paragonimus uterobilateralis*. American Journal of Tropical Medicine and Hygiene. 1983; 32(2):371–375. https://doi.org/10.4269/ajtmh.1983.32.371 PMID: 6837846

101. Moyou-Somo R, Enyong PA, Kouamouo J, Dinga JS, Couprie B and Ripert C. Etude de la paragonimose dans cinq départements de la Mémé (Sudouest du Cameroun). Résultats du traitement par le praziquantel. Revue Science et Technique, Yaoundé. 1983; 6–7:125–129

102. Ochigbo SO, Ekanem EE, Udo, JJ. Prevalence and intensity of *Paragonimus uterobilateralis* infection among school children in Oban village, South Eastern, Nigeria. Tropical Doctor. 2007; 37(4):224–226. https://doi.org/10.1258/004947507782332766 PMID: 17988485

103. Osoagbaka OU, Njoku-Obi ANU. Bacterial Etiology of Lower Respiratory Tract Infections in parts of Eastern Nigeria. Nigerian Journal of Microbiology. 1982; 2:131–140.

104. Njunda AL, Nsagha DS, Assob JCN, Abange BW, Tamoh AJ, Kwenti TE. Pulmonary paragonimiasis and aspergillosis in patients suspected of tuberculosis in Yaounde, Cameroon. British Microbiology Research Journal. 2015; 10(4):1–8. https://doi.org/10.9734/BMRJ/2015/20138

105. Sachs R. Occurence of Human Lung Fluke Infection in an Endemic Area in Liberia. In: Geerts S, Kumar V, Brandt J. (eds) Helminth Zoonoses. Current Topics in Veterinary Medicine and Animal Science, Springer, Dordrecht. 1987; 43: 132–136. https://doi.org/10.1007/978-94-009-3341-5_19

106. Sachs R, Jarrett B, Attia B. Paragonimiasis and tuberculosis in the Bong area. Annual Report 1985, Liberia Research Unit of the Tropical Institute Hamburg 1985a; p43

107. Adedoyin MA, Awogun IA, Juergensen T. Prevalence of intestinal parasitoses in relationship to diarrhoea among children in Ilorin. West African Journal of Medicine. 1990; 9(2):83–88. PMID: 2268573

108. Yamamoto H, Aanu NO, Chukwuma F, Ugwuegblam I, Ahumibe E. Parasitological Surveys in The East Central State Of Nigeria. Japanese Journal of Tropical Medicine and Hygiene. 1979. 7(1):7–21.

109. Onubogu UV. Intestinal parasites of school children in urban and rural areas of Eastern Nigeria. Zentralblatt fur Bakteriologie, Parasitenkunde, Infektionskrankheiten und Hygiene. Erste Abteilung Originale. Reihe A: Medizinische Mikrobiologie und Parasitologie. 1978; 242(1):121–132.

110. Voelker J, Nwokolo C. Human paragonimiasis in eastern Nigeria caused by *Paragonimus uterobilateralis*. Zeitschrift für Tropenmedizin und Parasitologie. 1973; 24(3):323–328. PMID: 4744219

111. Dierckx P, Coigneau H, Debondt R, Leblanc G. Paragonimose pulmonaire. Annales de la Société Belge de Médecine Tropicale. 1980; 60:111.

112. Petavy AF, Cambon M, Demeocq F, Dechelotte P. Un cas Gabonais de paragonimose chez un enfant. Bulletin de la Société de Pathologie Exotique et de ses filiales. 1981; 74(2):193–197. PMID: 7249244

113. Simarro PP, Alamo A, Sima FO, Roche J, Mir M, Ndong P. Endemic human paragonimiasis in Equatorial Guinea. Detection of the existence of endemic human paragonimiasis in Equatorial Guinea as a result of an integrated sanitary programme. Tropical and Geographical Medicine. 1991; 43(3):326–328. PMID: 1816673

114. Aka NA, Assoumou A, Adoubryn KD, Djino S, Domoua K, Ouhon J, Kouassi EB, Rondelaud D, Dreyfuss G. Un nouveau foyer de paragonimose humaine découvert en Côte d'Ivoire (Afrique de l'Ouest): le cas de l' île de Lauzoua. Médecine tropicale. 2009; 69(3):263–266. PMID: 19702148

115. Aka NA, Assoumou A, Adoubryn KD, Djino S, Domoua K, Ouhon J, Kouassi EB, Rondelaud D, Dreyfuss G. Persistance d'un foyer de paragonimose dans le département de Lakota, Côte d'Ivoire (Afrique de l'Ouest). Bulletin de la Société de Pathologie Exotique et de ses filiales. 2008b; 101(5):407–409. PMID: 19192612

116. Eke RA, Enwereji EE. Re-Emergence of Paragonimiasis in Nigeria: A Case Report and Future Challenges. TAF Preventive Medicine Bulletin. 2010; 9(6):703–706. https://doi.org/10.5455/pmb.20100427030344

117. Ekanem AM, Oloyede IP, Eduwem DU. Paragonimiasis: A knowledge and awareness survey of clinicians in southern Nigeria on a neglected tropical disease. IBOM Medical Journal. 2020; 13(2):110–119.

118. Dou G, Aka N, Kouadio-Yapo C, Zika K, Loukou K, Ouhon J, Assoumou A, Adoubryn K. Connaissances Des Medecins Des Services De Pneumologie D'Abidjan Sur La Paragonimose. Revue Bio-Africa. 2018; 17:9–13.

119. Nkouawa A, Sako Y, Itoh S, Kouojip-Mabou A, Nganou CN, Saijo Y, Knapp J, Yamasaki H, Nakao M, Nakaya K, Moyou-Somo R, Ito A. Serological studies of neurologic helminthic infections in rural areas of southwest Cameroon: toxocariasis, cysticercosis and paragonimiasis. PLoS Neglected Tropical Diseases. 2010; 4(7):e732. https://doi.org/10.1371/journal.pntd.0000732 PMID: 20625553

120. Nkouawa A, Sako Y, Moyou-Somo R, Ito A. Serological and molecular tools to detect neurologic parasitic zoonoses in rural Cameroon. Southeast Asian Journal of Tropical Medicine and Public Health. 2011; 42(6):1365. PMID: 22299404

121. Bakuza J. Epidemiological Significance of Parasitic Infections in Olive Baboons (*Papio anubis*) at Gombe National Park, Tanzania. Tanzania Journal of Science. 2020; 46(1):137–150.

122. Sachs R, Cumberlidge N. The dwarf river crab *Liberonautes latidactylus nanoides* Cumberlidge & Sachs, 1989, from Liberia-a new second intermediate host of

Paragonimus uterobilateralis. Tropenmedizin und Parasitologie. 1990b; 42(1):73–74. PMID: 2052862

123. Arimoro FO, Orogun EO. Notes on the biology and ecology of *Sudanonautes floweri* (De Man, 1901; Crustacea: Brachyura: Potamoidea: Potamonautidae) in river Ogbomwen, southern Nigeria. Acta Biologica Colombiana. 2008; 13(1):65–78.

124. Ogban E, Ojukpong GK, Uttah EC. Assessment of metacercariae infection of edible crabs in an area endemic for paragonimiasis in the Cross River Basin. Pacific Journal of Medical Sciences. 2018; 19(1):30–40.

125. Sachs R, Cumberlidge N. Distribution of metacercariae in freshwater crabs in relation to Paragonimus infection of children in Liberia, West Africa. Annals of Tropical Medicine and Parasitology. 1990c; 84(3):277–80.

126. Sachs R, Cumberlidge N. Metacercarial load of fresh-water crabs *Liberonautes latidactylus* in an endemic paragonimiasis focus in Liberia, West Africa. Zeitschrift für Angewandte Zoologie. 1991b; 78:161–165.

127. Sachs R, Cumberlidge N. First record of the spiny river crab, *Liberonautes chaperi*, A. Milne Edwards, 1887, as second intermediate host of *Paragonimus uterobilateralis* in Liberia. Annals of Tropical Medicine & Parasitology. 1991c; 85(4):471–472. https://doi.org/10.1080/00034983.1991.11812595 PMID: 1796892

128. Fa JE, Seymour S, Dupain JE, Amin R, Albrechtsen L, Macdonald D. Getting to grips with the magnitude of exploitation: bushmeat in the Cross–Sanaga rivers region, Nigeria and Cameroon. Biological Conservation. 2006; 129(4):497–510.

129. Cabaret J, Bayssade-Dufour C, Tami G, Albaret JL. Identification of African Paragonimidae by multivariate analysis of the eggs. Acta Tropica. 1999; 72(1):79–89. https://doi.org/10.1016/s0001-706x(98)00074-6 PMID: 9924963

130. Aka NA, Assoumou A, Adoubryn KD, Djino S, Ouhon J, Rondelaud D, Dreyfuss G. Presence of three egg groups of Paragonimidae in South-Western Ivory Coast (West Africa). In: Dupoy-Camet J, Dei-Cas E. (eds.) X European Multicolloquium of Parasitology. EMOP free papers. Paris, August 24–29 2008. Publ. Medimond, Bologna. 2008; 195–198.

131. Nozais JP, Doucet J, Dunan J, Assale N'dri G. Les paragonimoses en Afrique Noire. Apropos d'un foyer recent de Cote d'Ivoire. Bulletin de la Société de Pathologie Exotique et de ses filiales. 1980; 73(2):155–163. PMID: 6257410

132. Adoubryn KD, Ouhon J, Assoumou A, Kassi EA, Koné M, Therizol-Ferly M. Champignons et parasites isolés à l'examen de 142 liquides d'aspiration bronchique à Abidjan (Côte d'Ivoire). Médecine d'Afrique Noire, 1999; 46(7):362–365.

133. Traoré SG. Risques de contraction des affections à Vibrio sp. et à Paragonimus sp. liés à la consommation des crabes et des crevettes vendus sur les marchés d'Abidjan et de Dabou. Doctoral dissertation, Universite Nangui-Abrogoua. 2013. pp. 198

134. Vandepitte J, Job A, Delaisse J, Tabary M. Quatre cas d'abcès rétroauriculaires chez des Congolais, produits par un nouveau distome. Annales de la Société Belge de Médecine Tropicale. 1957; 37(2):309–316. PMID: 13459039

135. CILSS Landscapes of West Africa—a window on a changing world. Ouagadougou, Burkina Faso. U.S. Geological Survey EROS, 47914 252nd St, Garretson, SD 57030, United States. 2016; pp. 219.

136. Ikhuoriah SO, Awharitoma AO. Prevalence of parasitic infestation in freshwater crab, *Sudanonautes africanus* (A. Milne-Edwards, 1869) (Brachyura: Potamonautidae) from selected Rivers in Edo and Delta States of Nigeria. Journal of Applied Sciences and Environmental Management. 2018; 22(11): 1761–1767. https://doi.org/10.4314/jasem.v22i11.9

137. Kaze PD, Gam KP. Survey of Cysticercus (Bladder Worm) in Meat Sold for Consumption in Bukuru, Plateau State Nigeria. Academic Journal of Interdisciplinary Studies. 2013; 2(12):45–50. https://doi.org/10.1038/s41437-017-0035-0 PMID: 29279603

138. Demenou BB, Doucet JL, Hardy OJ. History of the fragmentation of the African rain forest in the Dahomey Gap: insight from the demographic history of *Terminalia superba*. Heredity. 2018; 120(6):547–561. https://doi.org/10.1038/s41437-017-0035-0

139. Gouws G, Stewart BA. Potamonautid river crabs (Decapoda, Brachyura, Potamonautidae) of KwaZulu-Natal, South Africa. Water SA. 2001; 27(1):85–98. https://doi.org/10.4314/wsa.v27i1.5015

138. Sachs R, Kern P, Voelker J. Le *Paragonimus uterobilateralis* comme cause de trois cas de *Paragonimose humaine* au Gabon. Tropenmedizin und Parasitologie. 1983; 34(2):105–108. PMID: 6879703

140. Sachs R, Kern P, Voelker J. *Paragonimus uterobilateralis* as the cause of 3 cases of human paragonimiasis in Gabon. Tropenmedizin und Parasitologie. 1983; 34(2):105–108. PMID: 6879703

141. Rabone M, Allan F, Gouvras AN, Pennance T, Hamidou A, Webster BL, Labbo R, Emery AM, Garba AD, Rollinson D. Freshwater snails of biomedical importance in the Niger River Valley: evidence of spatial and temporal patterns in abundance, distribution and infection with *Schistosoma* spp. Parasites & Vectors. 2019; 12(1):498. https://doi.org/10.1186/s13071-019-3745-8 PMID: 31640811

FIGURE CAPTIONS

Fig 1. Flow diagram for the selection and assessment of studies for the systematic review of paragonimiasis and *Paragonimus* spp. in Africa, and the subset of *Poikilorchis congolensis*

Fig 2. Total number of studies by decade and country. Publications of experimental studies (1970s and 1980s) or case reports where the origin of infection could not be ascertained (1940s, 1960s) are shown in grey (country = NA). For a longitudinal study, final decade only is shown (**88**).

Fig 3. P*aragonimus* spp. and *Poikilorchis congolensis* egg length and width measurements in µm, combined data from 37 studies in total.

Fig 4. Freshwater crab host occurrences by species. 1970- 2017, Africa wide, and West and Central African distributions (crab occurrence data combines both unpublished dataset from author NC, and records from literature via systematic review).

Fig 5. Occurrences by species: *Paragonimus* spp. and *Poikilorchis congolensis* distribution from confirmed occurrences in the literature, 1910s- present (1919-2017). (A) West and Central Africa, (B), close-up to the main endemic region, the southeast Nigeria/Southwest Cameroon focus in the Cross River Basin, showing the Imo River and Cross River systems. Localities with very low coordinate uncertainty (from crab or mammal hosts are shown with red outlines

S Fig 1. Freshwater Crab host occurences by genus, 1970- 2017, from Cote d'Ivoire to South Sudan, and *Liberonautes* from Senegal to Ghana.

S Fig 2: *Paragonimus* occurrences by decade, 1910s- 2010s (1919-2017). Main endemic foci in Southeast Nigeria, Southwest Cameroon show evidence of ongoing infection spanning decades. Another key focus of infection in Liberia has not been surveyed post the 1980s as shown. No recent case occurences were evident in the literature for DRC and Republic of Congo, as shown.
