# Supplementary material for: Endemicity of Paragonimus and paragonimiasis in Sub-Saharan Africa: A systematic review and mapping reveals stability of transmission in endemic foci for a multi-host parasite system
Source: PLoS Negl Trop Dis. 2021 Feb 5;15(2):e0009120. doi: 10.1371/journal.pntd.0009120 (PMC7891758; doi:10.1371/journal.pntd.0009120)
Supplement: Attachment — Submitted filename: Letter_to_Reviewers_2020-11-11.docx [file pntd.0009120.s010.docx]

11^th^ November, 2020

Dear Editors Dr Blair, Dr Sripa, and Reviewers,

Thank you very much for a detailed, constructive and speedy review. We are very grateful for the suggestions, identification of omissions and points to address, and supportive comments.

Please note all line no references to the revision (as opposed to the original submission) are referencing the clean revision not the marked up/track change version. All line numbers for the revision are noted as such so it is clear they don’t refer to the line numbers of the original submission.

We would like to thank you all for your attention, this comprehensive review has substantially improved the manuscript.

**Editorial comments**
A few comments from myself. The geographical scope of the review is stated to be West and Central Africa. However, the rather small number of records from South Africa and e.g. Tanzania have been mentioned in the text. I wonder if the scope of the review should extend to all of Sub-Saharan Africa? Or even just "Africa", given that a record from Libya is mentioned.
Thank you for the suggestion. We have changed the title and the conclusion to reflect this. In terms of the contributions we have made, they are primarily for West and Central Africa. For elsewhere in Africa, for example, the previous review by Appleton et al for South Africa that also includes the grey literature, this work has previously already been done (why we have emphasised West and Central Africa).

In lines 41 and 71, "systemic" should read "systematic".

We had originally used systemic rather than systematic to avoid any implication that underreporting was deliberate, we have now removed both mentions of systemic.
Line 771. How certain are you that the report of Paragonimus from pigs in Ghana is credible? The identification was based only of the presence of trematode eggs in pig feces.

Regarding the report from pigs in Ghana, this assessment as ‘confirmed’ is consistent with the other assessments of the literature, those based on stool screens only we also examined photos where available and positive identifications of other trematodes (detailed in line 240-3 of the clean revision). We have since reread the publication and contacted the authors, and although egg morphology in microscopy photos were not available, we are satisfied it is a robust identification. We recognise it is an outlier however given the ecology of the region. Also it is in livestock, and a pig, not previously reported as a host in Africa (although we found another report from pigs in a separate publication, Kaze & Gam, 2013, but here assessed as an ‘unconfirmed’ case as there was not enough information). Given that sampling was undertaken in villages, we think highly likely pigs were foraging in the villages/surrounding regions and change of outside-country feed was very low. We have now flagged these points and mentioned it is an outlier in the discussion on line 800-1 of the clean revision.
Line 829. Check spelling of snail taxon names.

Thank you and apologies for the errors, we have now fixed snail taxon spellings
As one of the reviewers pointed out, Scientific names must be in italics, That includes in the reference list!

Thank you, we have now consistently italicised scientific names in the references

**In line comments: Reviewer 1**

Many thanks for the detailed comments and suggestions. These have been very helpful in improving the manuscript and we have incorporated most of them. We think the revision is much improved on the original submission as a result. Please note all line no references to the revision (as opposed to the original submission) are referencing the clean revision not the marked up/track change version.

Line 34: The abstract is detailed we agree but within the word limit, and as PLOS NTDS also provides author summaries- much reduced and simplified, we think it best to leave the abstract as is. We have made a few minor modifications to the author summary to simplify it further.

Line 90: global distribution- this is highlighted but no comment as added.

Line 97- we have now added an example of paratenic hosts on line 95 of the clean revision

Line 100: only species and genus names are italicised as per rules of nomenclature

Line 102: Brachyuran is highlighted but not comment is made?

Line 109: we have rephrased the sentence and the following one, to improve clarity

Line 120- see above- family name so not to be italicised

Line 197: this is highlighted but no comment? We have now moved this section to the introduction, line 137-149 of the revision.

Line 213: Most of the non-English papers were in German, translated by 2^nd^ author Joris who is a native speaker, or in French, I have a relatively sound working knowledge of written French so was able to translate much of the text directly, and use google translate where necessary. Google translate was used for the 2 Italian papers and the Dutch paper, I do not have a Dutch or Italian colleague to ask to translate so it seemed a viable alternative. We agree that it has limitations, but is widely used in systematic reviews, and was used on a relatively limited number of papers. We have now added usage of google translate as a limitation of the study section- line 878 of the clean revision.

line 226: You note this is not clear and seems like a leading statement, we do not follow what you mean here? We are outlining the epidemiological data that we recorded. Is the reference to leading statements meant for the next highlighted section- line 232 perhaps?

Line 232: this is highlighted but no comment is made. We guess you question the estimation of study period. This was problematic as a significant number of papers did not state survey period, but it did not seem viable to exclude them from the review merely for that reason especially given that the literature is relatively sparse as a whole. Examining the published literature, we came up with 2 years prior to publication as a reasonable estimate for when the study took place. Comment- ‘what effect does this have on the reported prevalences and the mapping data?’ we attempted to mitigate any bias as far as possible by examining temporal trends by decade only which we founds as a result was minimal- we have mentioned in the study limitations section.

Line 239: confirmation of infection- thank you for the suggestion, we agree that this is potentially misleading and have changed as suggested to ‘methodological assessment’ on line 235 of the revision.

Line 240 on: these are results/discussion- we agree, there is some overlap of methods and results which is why these sections were here in methods originally. We have moved to results section “species” line 390 onwards in the revision.

Line 276: suggest move to methodological assessment- thank you for the suggestion, we have moved, and added 2 subheadings under methodological assessment- ‘Identification’, and ‘assessment of surveys’, line 236, and line 246 respectively of the revision.

Line 289: suggest move section on assessment of age categories to results: we have done so, line 549-553 of the revision.

Line 302-310: suggest move to results, which we have done, line 501-3, and line 597-8 respectively, but for line 306-310 of the original submission, we have reworded to remove any reference to findings and left in methods as this is stating methods, this is in line 283-5 of the clean revision. We have also checked through the materials and methods section and any references to findings have been removed, eg “none were found” in line 262 of the original submission.

Line 399: Thank you for the observation, we have attempted to simplify text throughout and reword for clarity, however we have left the text description of the tables, to make inclusive for a range of readers, those who will focus on the tables and figures, and those on the text itself. We think it is also important to state the findings presented in the tables and figures.

Line 405: this is already stated on line 402-3 of the original submission. we mention again ‘reference lists as above’ in relation to ‘other sources’ on line 376 of the clean revision so there is no possibility of confusion.

Line 437: we have replaced includes with included and rephrased “survey” to “this systematic review” on the same line so it is clear we are talking about the present study and not the surveys themselves.

Line 554-557: we have rephrased as suggested

Line 557: we did try a table here but it didn’t work as there is not enough information here to sustain a standalone one. We agree clinical findings are of critical importance, but this was small section of the work which was not the main focus of the ms (or our area of expertise).

Line 594: thank you we have fixed spelling of hemoptysis

Line 592: we have fixed spelling of Bithionol

Line 605: We have rephrased the statement

Line 628: we have rephrased and removed the mention of low certainty, line 624-6 of the revision

Line 638: we have removed the hyphenation and rephrased so is clear we are referring to either crab hosts or wild mammal hosts only

Line 666: Thank you very much for this helpful suggestion, we have moved line 868-880 to the beginning of the discussion, line 640-52 in the revision, and added a subheader ‘summary of key findings’.

Line 684: the dosage of praziquantel as reported as effective in treating paragonimiasis in the reviewed papers and the dosage for schistosomiasis overlaps, we think there is a possibility that schistosomiasis treatment may also be treating paragonimiasis but to our knowledge there is no published data on this. We agree this could be a contributing factor in the ongoing neglect of paragonimiasis. This is something we would like to examine further at a later stage, for example with country Ministry of Health records.

Line 706: we agree and have removed the reference to zoonotic transmission as this is confusing the point of the sentence- here we want to highlight the fact that paragonimiasis in crab-eating mammal populations such as this olive baboon troop are showing signs of significant disease burden and paragonimiasis is potentially a wildlife disease of importance in Africa. We have also added a separate mention of the potential role of primates as sylvatic hosts as think this is an important point to address, referencing Friant et al., 2015 (ref. no. 39), line 691-5

Line 712: Thank you and we agree that the sentence is confusing, we have rephrased and split into 2 sentences, line 700-3 of the revision.

Line 748: this is a rather tricky concept to express in words, we have rephrased and moved this sentence so it directly follows the one “alternatively cercariae may leave an infected snail”, line 733-5 of the revision.

Line 793: Thank you we have added a sentence break as suggested, line 780-4 of the revision.

Line 837: Thank you very much for the astute assessments of the study limitation section. We agree this statement reflects gaps inherent in the literature but this is not a limitation of our review itself, however feel it is still important to flag. We have moved to further on in the paragraph- in context of not utilising MOH records (likely particularly useful for countries like DRC where no survey data are available. We have also substantially remodelled this paragraph to address limitations in our approach, added other limitations- eg usage of google translate, and made clear where we are specifically addressing characteristics of the literature itself, rather than our analysis thereof. Please see line 829-59 of the revision.

Line 883: here we are trying to address future directions and provide suggestions/thoughts for future work. We do not want to overcomplicate things by introducing new concepts or including a lengthy description of metabarcoding here (or bayesian modelling) but think it is important to flag these areas for further studies. We have rephrased to add context: “molecular methods such as metapbarcoding or environmental DNA sampling” and moved the other mention of metabaroding- as a technique to identify potential diet items therefore potential snail species to follow on from this, line 863-9 of the revision.

References: Notes on refs in general

1.Thank you for the careful checking, we have now italicised species names consistently throughout the references

2. Thank you again for checking, these references are respectively, a book, and 2 articles (the latter being in literature- a compendium of articles in a report), therefore in the latter 2 cases they are correctly formatted and in the former, the book, we have revised the reference.

3.62 and 84 are 2 articles within the same volume- it is a ‘grey’ literature publication – as above, a compendium of articles in a report (Annual report 1985: Liberia Research Unit of the Tropical Institute Hamburg). These are definitely separate articles with slightly different authorship within the report.

4. we have added the web address for Bakuza, 2012. In general is was very difficult to source thesis copies, other than the few publicly available (we tried various methods, contacting the libraries, interlibrary loans etc).

5. Thank you for spotting the extra reference – no 138 below 139 was originally added in error. We have also double checked all the references.

**General review comments**

METHODS:

Reviewer 1:

I question the legitimacy and validity of Google translation services as a robust tool for the translation of articles not presented in English, and lacking a knowledgable translator. For many languages, particularly of marginalised populations, this is not a reproducible or reliable translation tool.

A number of sections in the methods seem to be reporting results/ findings from the preliminary literature search and even the contents thereof. Authors should consider streamlining the presentation of methods alone and leave presentation of results to the appropriate section, in a systematic manner consistent with the methodology. Authors should consider presenting the methods as concisely as possible (probably with mention of deviations from the standard approach) and avoid including results or explanations thereof (discussions) within the methods section.

It is my opinion that following PRISMA guidelines, the authors have declared sufficient detail on selection and inclusion criteria. There is a bit of confusion in the statement/ explanation of the inclusion and exclusion criteria of articles, but this is mostly a problem of presentation.
The limitations of the study mentioned however, appear to apply more to the study reports reviewed and not specifically apply to this review. Key biases like volume and density of reporting, databases used to search for appropriate literature, and the inferences made from the meta-analysis done could improve the quality and confidence in the findings of this study

Thank you for the very helpful assessment and insightful comments. We have addressed the concern on usage of Google Translate, please see as detailed for in line comment responses above, and now added as a study limitation in the relevant paragraph in the discussion. Thank you for identifying the confusion of results in the methods section, some of the methods were certainly overlapping results. We had originally added reference to some studies to help describe selection criteria but thought also at the time that this was potentially an issue as included results. Now we have removed references to findings within the methods, please see as detailed in relation to ‘in line comments’ above. We have attempted to simplify and clarify wording throughout. For example we removed the reference to other diseases surveyed in the subset analysis section (line 470-2 or the original) as this was potentially confusing with the latter section on ‘other diseases surveyed’ (line 595 of the revision); and reference to finding of prevalence in *Sudanonautes aubryi* as the sample size here was very small (line 522-525 of the original submission). We have not substantially shortened the results text as believe it is important to have text as well as figure and table representation of the results to allow for different readership styles and to make sure there is text representation of all results presented (please see above).

We thank the reviewer for the astute points on the study limitations section. We have substantially rewritten this section, clarifying what is referencing characteristics of the literature itself (where necessary to mention), and added to limitations of our analysis/ systematic review methods. We did go to considerable lengths to source all relevant literature, and do not see likely bias at study selection stage eg in terms of databases used, but, we do think there is significant potential relevant grey literature, Ministry of Health records and similar that we did not include in the search. Given the large number of historical papers here, and the large number only available by interlibrary loans, this review already involved significant work however to find the published literature, without including the grey literature. We do however as stated in the limitations section see this as a valuable source of information, and a potential source of bias in its exclusion (especially for those countries with sparse literature like DRC) and worth following up at a later stage.

Reviewer #2: The objectives are clearly articulated with a clear testable hypothesis. The geographical coverage and the sample size is large which allows for the generation of robust scientific data.

Thank you very much for the observation, we had hoped to achieve a wide coverage with the review.

Reviewer #3: **no**major new analyses/experiments require

Reviewer #4: -Are the objectives of the study clearly articulated with a clear testable hypothesis stated? Yes, they are
-Is the study design appropriate to address the stated objectives? Yes
-Is the population clearly described and appropriate for the hypothesis being tested? NA
-Is the sample size sufficient to ensure adequate power to address the hypothesis being tested? NA
-Were correct statistical analysis used to support conclusions? Yes
-Are there concerns about ethical or regulatory requirements being met? No

RESULTS

Reviewer #1: Analysis presented does meet the requirement stipulated in the design. Generally results seem to be over detailed and contain very lengthy narratives that are quite difficult to follow. Authors should consider using short clear statements and present related results/ metrics together.

Results appear to be jumbled up in presentation from as early as multiple sections of the methods (239-262; 289-292; 302-310), as well as content more appropriate to the discussion or introduction (e.g. lines 197 - 206). Also, discussion of results should be left to the appropriate section as this makes the narrative lengthy and difficult to follow.

Some critical results e.g. common clinical findings/ presentations of paragonimiasis do warrant clear and independent presentation e.g. in a table, as this makes the information easier to appreciate.

Some of the figures (e.g. Fig 3) could benefit from better descriptive legends and some of the key statistical information presented.
Tables are quite detailed and presentable; therefore, some of the information presented in the results text is redundant and makes the paper somewhat over detailed and technical. Authors could consider watering down the results narrative to only key/critical findings, and refer readers to the tables for more detailed information.

We are thankful for the very helpful feedback. We have attempted to clarify narratives throughout, breaking up long sentences, and restructuring where necessary

We considered the suggestion to add the clinical findings into a table, and agree clinical findings are very important, but as mentioned above, we attempted to construct one but there are not adequate data here to sustain a separate table, this was comparatively a very small section of the review and not the focus of the manuscript.

We certainly agree it is a very lengthy and detailed manuscript but to a degree this is necessitated by the amount of information we are trying to convey- a direct result of the type of manuscript that it is, a 2 part- systematic review and mapping publication, requiring description for all relevant sections- whether methods, results or discussion. We have decided to leave the majority of the results text intact, to give readers the option to study the tables and figures, and or read the text, and think it is important to have text representation of table 1 for example. We acknowledge this may make it a rather long text to read and digest, but hope we have accounted for this in production of several figures, tables and the short summaries. Regarding figure legends: we have reviewed the figure 3 legend, tweaked all to make more informative and added missing legends for the supplementary tables (provided as excel files).

Reviewer #2: The data has been well analysed using appropriate statistical test. Overall, the results are well presented in line with the specific objectives.Tthank you for your comment
Reviewer #3: Table 1 appears to be missing
Reviewer 3: Apologies Table 1 was in the original submission (at the very end of the manuscript after the figures). It is now incorporated into the main manuscript after the references rather than a separate file.
Reviewer #4: -Does the analysis presented match the analysis plan? Yes
-Are the results clearly and completely presented? Yes
-Are the figures (Tables, Images) of sufficient quality for clarity? Yes, they are

Many thanks

Reviewer #1: Some confusion/ misrepresentation of discussion/ result material within the conclusion section (868-880). Overall conclusions are based on key findings from the study and provide useful insights into the future considerations and implications of the findings.

Good emphasis of public health relevance as well as policy and intervention plans are laid out in the conclusion section of the paper
Thank you for a very helpful suggestion. We have moved the summary of key findings to the beginning of the discussion (line 868-880 of the original submission). This suggestion has improved both sections and made the conclusion more cohesive and focussed as a result.

Reviewer #2: The conclusions are accurately supported by the data generated.
Thank you

Reviewer #3: The conclusions are clearly supported with the data and the authors describe the limitations of their data set. The significance of their findings to understanding the African paragonimiasis and public health relevance us adequately adressed.
Thank you

Reviewer #4: -Are the conclusions supported by the data presented? Yes
-Are the limitations of analysis clearly described? Yes
-Do the authors discuss how these data can be helpful to advance our understanding of the topic under study? Yes they do
-Is public health relevance addressed? Yes it is

Thank you

CONCLUSIONS:

Editorial and data presentation modifications

Reviewer #1: The paper is generally of significant importance to the field and addresses a highly neglected trematodiasis on the continent;
1. Authors need to revise the language and sentence structure in a number of places. Use short and clear statements/ sentences especially when presenting complex concepts.

Thank you very much for the feedback. We have attempted to simplify and shorten sentences throughout, please see track change version, and also specific examples as above.
2. Consistency in presentation e.g. of non-English terminologies (with correct spellings every time e.g line 582 and related references to hemoptysis; line 592 and further mentions of Bithionol). Rules of consistent italicising scientific/ classification names should also be revisited

Thank you for noticing the errors, we have fixed these typos. For italicisation of scientific names, we have followed standard scientific nomenclature rules, that only species and genus names are italicised, not family or superfamily names
3. Some general restructure of the methods, results and discussion sections should help make the paper easier to read and especially highlighting of key points for non-technical audiences.

Thank you for the constructive comments and as detailed in the ‘in line comments’ above, we have addressed the issues and restructured these sections.
4. Thorough review and revision of the references (noting repetitions, numbering, formatting of references). Since this is a review of the literature, the references section indeed counts as the raw data/ results from the study and should be robustly presented.
We agree the references section (and the supplementary tables with the full papers list) count as raw data/results, and it is particularly important to make sure these are correct. We have double checked/cross checked all references amending where necessary (removal of the extraneous reference- Sachs et al. 1983, no 138 in the original submission, replacement of Plouvier et al with Loubiere et al, 1976, renumbering of references after the restructuring of methods/results/discussion as outlined above.

Reviewer #2: Results:
• Lines 408 – 411: The number of studies doesn't add up to the totals. If you add up 132, 3, 2, 3, 4, it gives 144 and not 142 scientific publications as stated. There is also a consensus with figure 1 that indicates 149 studies on paragonimiasis, were included. And if you add to the 7 articles on Poikilorchis congolensis, it gives 156.

Thank you very much for the careful checking of the totals and apologies for the errors. We have fixed these errors in the text and the figure (please note also we received an outstanding interlibrary loan post submission so the total is now 143 as referenced also in the main text). Please see the amended fig 1.
• Lines 611 – 615: Correct the spellings of the following organisms: Trichuris trichiura, Strongyloides stercoralis, Enterobius vermicularis. Verify the spelling of all other scientific names in the manuscript.

Many thanks again for spotting the errors, and apologies, we have now fixed spellings of the STHs in the paper and checked all scientific name spellings throughout.
• Figure 3: Include the units of measurement in the Y-axes of the two graphs.

Thank you for spotting the omission, we have now added units of measurement.
• Figures 3 and 5: Kindly improve on the resolutions of these figures; the labels are barely visible.

Figure 3 and 5: the full resolution figures are available as a download link, we have also attempted to improve resolution of the compressed files and have made minor adjustments to improve the figures generally.
• Tables 1 and 2: kindly move the keys to the bottom of each table.
The journal guidelines for PLOSNTDs requires the keys at the top of the table so we have left them at the top.
Discussion:
Lines 680-682: Refs 104 also reports on paragonimiasis and tuberculosis co-infection.

Thank you for the observation, yes indeed Njunda et al surveyed both TB and paragonimiasis as referenced on line 603 of the original submission and in line 599 of the revision, and in the discussion in relation to paragonimiasis in Cameroon on line 657 of the revision, but did not state reports of any co-infections (ie any individuals with both diseases) which we specifically address in this point of the discussion.

Reviewer #3: Minor edits:
109: Space missing between 3 and decades
Line 109: we have added the missing space

116: Do you mean transmission “within” Nigeria?

Line 116: Thank you we have replaced ‘from’ with ‘within’
129: P. gondwanensis and P. keberti are mentioned within the introduction and within results, but little information is provided on these putative (?) species. It would be helpful to have a little more background on how these are differentiated from the two main species, africanus and uterobilateralis. Some discussion on the reasoning for differentiating these species and the validity of this distinction would be helpful in the discussion.

Line 129. Many thanks we agree that these are putative species and had a section in an earlier version discussing this point, we have now added reference to *P gondwaniensis* and *P. kerberti* in the introduction to the effect that they should be regarded as unconfirmed, being published without molecular data (this is particularly notable given how recent the publications are).

We have also mentioned this now in study limitations as a potential bias to analyse species separately given that these should be regarded as putative species although are presently named species, line 837-9 of the clean revision
150: A brief introduction to Poikilorchis congolensis as related to Paragonimus spp. within the introduction would be useful. It is not immediately clear why it is included.

Line 150: Thank you for the helpful suggestion, we have now added a short paragraph on *Poikilorchis congolensis* into the introduction (including some text originally in the methods), line 140-8 in the clean revision
166: It is very clear how the human data and intermediate host data were handled for prevalence, intensity, mapping etc., but there is limited information on how data from other mammalian hosts were handled. Where missing, please include details on when they were excluded from analysis and why, or when they were included and how?

Line 166: Many thanks for the observation and suggestions and for spotting these omissions. Apart from the few systematic mammalian surveys referenced, most sampling of mammals was relatively sporadic and opportunistic in nature, eg if researchers came across scats, e.g. no ecological sampling, transects etc. In some earlier cases, mammals were sacrificed and screened for worms in autopsy, by authors Sachs and Voelker in particular. (NB in the discussion we mention that non-invasive faecal sampling only should be utilised in further mammal sampling). In general given that most of the mammal surveys were sporadic and sample sizes small, we made sure only refer to qualitative findings in the discussion. Only 16 the papers including data on mammals could be included in the prevalence table, e.g. the total number of animals surveyed was provided, and of these, only a few were systematic surveys (eg refs 39, 66, 81). We reread and revisited all the relevant publications, reran analysis and made some adjustments to the data table- S2 Table (now incorporating other data, including info on study assessment), and Table 1. We have now included description into results on line 477 to 484 of the revision, including qualitative observations on where surveys took place.
169: Briefly describe or at least spell out the PRISMA guidelines.

Line 169: Given that the methods are already lengthy, we have included the checklist- which outlines all guidelines, in supplementary files (S file 1).
202: The sentence beginning with “Symptomology” is long and somewhat confusing. Consider breaking this down into multiple sentences and referring specifically to P. congolensis for clarity.

Line 202 Thank you we agree the sentence was rather clunky and have rephrased and moved to introduction as suggested above, line 140-8 of clean revision.
220: Is this referring to S1 and S5 tables? If so, S5 should be re-named S2 since it is the second table referenced in the manuscript.
Line 220 apologies for the confusion, the S1 & 5 table was a typo, this has been amended to S1-5 tables as here we refer to all the tables, line 217 of the revision

309: The meaning of “…the levels reported were assessed in general observations” is not clear
Line 309 we have removed the mention of “the levels were assessed in general observations”

324: Site type: These describe human, crab, and snail sampling location. None of these seem to describe sites where samples form wild animals would have been obtained.

Line 324: Thank you for identifying this omission, we have now mentioned mammal survey sites in line 352-3 of the clean revision
330: Occurrences of crab intermediate hosts: would be helpful to have a brief description of this unpublished dataset, including how it was constructed. For example, were these extracted from the literature, based on niche modelling, from empirically collected data?

Line 330: Many thanks for noting the omission, the unpublished geolocated records of occurrence for the freshwater crab hosts in are based on empirically collected data from field research carried out by NC from 1980 to 1984 in Nigeria and Cameroon, and from 1988 to 1989 in Liberia. The other records from Nigeria, Cameroon and Cote d’Ivoire are from the literature (mostly the works of Voelker and Sachs and some recent works from various authors) and the other records from Liberia are from the literature (works by Sachs and Cumberlidge). We have added description to this effect to the manuscript, line 306-9 of the revision, and added into the figure legends for Fig 4 and S Fig 1. We have also now added the unpublished dataset into supplementary (S6 Table).
337: Unclear what is meant by the “subsequent source”
Line 337: Apologies, we removed mention of “subsequent source” as it was not needed

484: Suggest re-wording to enhance clarity. Include % prevalence for africanus in addition to uterobilateralis. Specify what the 3.4% refers to for Cameroon and what comparison is being made to produce the p-value.
Line 484: Thank you we have reworded to make clear that the 3.4% refers to *P. africanus* prevalence- and the comparison is by country- ie *P. uterobilateralis* for Nigeria only versus *P. africanus* for Cameroon, line 464-6 of the revision (P value from a Wilkoxon ranked p value, not stated here as mentioned in methods).

502: Some indication of variation in the 43.2% statistic would be informative (e.g. range or SD)
Line 502: Thank you for the helpful suggestion, we have added range to the prevalence value – line 487 of the clean revision and for the other prevalence values given in the paragraph (to line 496), and also the missing values for *Paragonimus* sp (ie species not identified) for Feliformia.

503: Provide incidence data from Feliformia and primates or remove.
Line 503: we removed reference to incidence data here- as we have not specifically referenced or discussed incidence in the ms.

544: Highlight here that no information was available for P. kerberti or gondwanensis if appropriate

Line 544: We agree it is appropriate to mention and have added that data for neither *P. gondwanensis* or *P. kerberti* is actually available, line 533 of the revision, and also into the legend for Fig 3.
705: How does emaciation and high intensity infection suggest zoonotic transmission? This link is unsubstantiated/ unclear.

Line 705: Thank you for the helpful observation, reviewer 1 made a similar comment, we agree this wording was confusing and potentially misleading even. The point of this paragraph was really to highlight that there is evidence (in a few papers) that paragonimiasis represents a significant wildlife disease (independent from any discussion on human infection). However, rather than remove all reference to zoonotic transmission, we think it is important to note also that primates and wildlife in general may play a significant role in zoonotic transmission, and reference Friant et al (39) in relation to the potential role of sylvatic hosts in human infection. We mention this also as a further area for study.
Figure 5: It is hard to tell the difference between kerberti and gondwanensis within the map, since they can often only be seen by partial circles. I suggest making the circles different colors.

Figure 5: Thank you we agree and we have altered the map, to have 2 different colours for the 2 species and to make the circles slightly larger (and fixed an error in the legend).

Reviewer #4: Line 38: Maybe instead of writing “Intermediate hosts: freshwater snails and crabs”, it was better just to write as in line 88, “primary snail host” since land snails were suspected to play a role by some authors; even though, this information is yet to be confirmed.

Line 38: Thank you for the astute observation. We agree it is problematic to state freshwater for the snail intermediate hosts given that the host is unconfirmed. We rephrased to “snails and freshwater crabs” in line 37 of the revision. For ‘primary’, we specifically chose to refer to 1^st^ intermediate host throughout rather than primary host to avoid any potential confusion with the definitive host.
Introduction: It seems very long. I suggest that the authors should delete some sentences and bring them into the discussion session.

We agree the introduction is long, however the paper as a whole is lengthy and the discussion is particularly long (we found it challenging keep it to a reasonable length). We thought on balance best to avoid shifting any sections to the discussion and have tried to improve the introduction to remove any discursive elements.

Summary and General Comments

Many thanks to all the reviewers for these supportive comments, and for the very detailed attention throughout which has significantly improved the manuscript. What we hoped to achieve was to address infection in all hosts, to highlight the many recent publications – a number of which have been overlooked by the international NTD community, to map distributions over time and to encourage further studies by providing a basis for further work for this particularly neglected disease.

Thank you so much,

Yours sincerely,

Muriel Rabone (on behalf of all the authors, Joris Hendrik Wiethase, David Rollinson, Paul Clark, Neil Cumberlidge, and Aidan Mark Emery)
