# Supplementary material for: Endemicity of Paragonimus and paragonimiasis in Sub-Saharan Africa: A systematic review and mapping reveals stability of transmission in endemic foci for a multi-host parasite system
Source: PLoS Negl Trop Dis. 2021 Feb 5;15(2):e0009120. doi: 10.1371/journal.pntd.0009120 (PMC7891758; doi:10.1371/journal.pntd.0009120)
Supplement: Attachment — Submitted filename: Letter_editors-reviewers_2020-12-30.docx [file pntd.0009120.s012.docx]

30^th^ December, 2020

Dear Editors Dr Blair, Dr Sripa and Reviewers 1 and 3,

Many thanks for the review of the revision and we are happy to hear the edits have been accepted. We have made a few very minor edits also- a few typos/very minor rewording needed- identified on rereading.

**Reviewer 1:**

Line 297: thankyou for identifying, the reference to Cumberlidge et al., 2018 has been reworded as follows “Locality data were also extracted from Table 2 of Cumberlidge et al., 2018 (Table 2; 46)”

Line 255: the tidyverse package is already cited, we recognise the point for R, but thought best to leave as is and the software and version are cited in the main text as is common practice.

**Additional minor changes**

Line 4: comma replaced with a colon

Line 69-71 minor rewording of ‘neglected tropical disease’ to ‘particularly neglected disease’- initial wording reads a bit oddly as paragonimiasis is an NTD in Asia too, and the point here is that it is particularly neglected in Africa. Replaced ‘where’ with ‘and’ as where is implicit

Line 82 extraneous comma- removed

Line 120 typo fixed- semicolon changed to comma

Line 186, 209 and 215 replaces author initials MR with MER so is consistent throughout ms

Line 284: typo fixed- extra comma and space removed

Line 398: missing space before ‘with’ added

Line 700: missing space added after ‘in’

Line 716: ‘different species’ was written in 2 subsequent sentences- rephrased the first to ‘crab species’ also so is clear we refer to the crab hosts here.

Line 768: typo fixed- comma replaced with semicolon

Line 805: Figs 4, 5 to figs 4-6 as all show sampling gaps

Line 843-6: is a bit of rewording here with the addition of ‘and the quantitative findings should duly be regarded as requiring confirmation in additional studies‘ - we felt it is necessary to clarify further that although we include some quantitative analysis that this is categorically not a meta-analysis and shouldn’t be interpreted as such, and the quantitative findings (eg higher *P. uterobilateralis* than *P. africanus* prevalence in humans) need to be qualified as such ie regarded as needing confirmation with additional field studies. apologies for more than a very minor change at this point but we think this addition is worth making.

Line 863: ‘significantly improved’ replaced with ‘strengthened’ – as is it not really the authors place to state whether it has improved or not but we are perhaps justified in stating it has been strengthened

Line 865: ‘sampling’ was repeated in error- duplicated word removed.

Line 899: updated missing author contributions for who conceived the study

Also: S Fig 2 changed to Fig 6: line 636, 784, and fig caption in line 1541-45. the temporal stability in the endemic zones is a main finding and referenced in the title so this should be in the main paper not supplementary- apologies for the late change.

Please also note that the unpublished dataset referenced in the manuscript (geolocated records of occurrence for the freshwater crab second intermediate hosts in West Africa) are included in Supplementary Table 6.

Yours Sincerely,

Muriel Rabone (on behalf of all the authors, Joris Hendrik Wiethase, David Rollinson, Paul Clark, Neil Cumberlidge, and Aidan Mark Emery)
